# A QTL on the short arm of wheat (*Triticum aestivum* L.) chromosome 3B affects the stability of grain weight in plants exposed to a brief heat shock early in grain filling

**DOI:** 10.1186/s12870-016-0784-6

**Published:** 2016-04-22

**Authors:** Hamid Shirdelmoghanloo, Julian D. Taylor, Iman Lohraseb, Huwaida Rabie, Chris Brien, Andy Timmins, Peter Martin, Diane E. Mather, Livinus Emebiri, Nicholas C. Collins

**Affiliations:** The Australian Centre for Plant Functional Genomics, School of Agriculture Food and Wine, The University of Adelaide, PMB 1, Glen Osmond, SA 5064 Australia; School of Agriculture Food and Wine, The University of Adelaide, PMB 1, Glen Osmond, SA 5064 Australia; Phenomics and Bioinformatics Research Centre, University of South Australia, GPO Box 2471, Adelaide, SA 5001 Australia; Present address: Mathematics Department, Bethlehem University, PO Box 11407, Rue des Freres, Bethlehem, 92248 Jerusalem Palestine; The Plant Accelerator, The University of Adelaide, PMB 1, Glen Osmond, SA 5064 Australia; EH Graham Centre for Agricultural Innovation, Pine Gully Road, Wagga Wagga, NSW 2650 Australia

**Keywords:** Heat tolerance, Wheat, *Triticum aestivum*, Quantitative trait loci, QTL, Stay-green, Senescence, Grain size, Grain filling

## Abstract

**Background:**

Molecular markers and knowledge of traits associated with heat tolerance are likely to provide breeders with a more efficient means of selecting wheat varieties able to maintain grain size after heat waves during early grain filling.

**Results:**

A population of 144 doubled haploids derived from a cross between the Australian wheat varieties Drysdale and Waagan was mapped using the wheat Illumina iSelect 9,000 feature single nucleotide polymorphism marker array and used to detect quantitative trait loci for heat tolerance of final single grain weight and related traits. Plants were subjected to a 3 d heat treatment (37 °C/27 °C day/night) in a growth chamber at 10 d after anthesis and trait responses calculated by comparison to untreated control plants. A locus for single grain weight stability was detected on the short arm of chromosome 3B in both winter- and autumn-sown experiments, determining up to 2.5 mg difference in heat-induced single grain weight loss. In one of the experiments, a locus with a weaker effect on grain weight stability was detected on chromosome 6B. Among the traits measured, the rate of flag leaf chlorophyll loss over the course of the heat treatment and reduction in shoot weight due to heat were indicators of loci with significant grain weight tolerance effects, with alleles for grain weight stability also conferring stability of chlorophyll (‘stay-green’) and shoot weight. Chlorophyll loss during the treatment, requiring only two non-destructive readings to be taken, directly before and after a heat event, may prove convenient for identifying heat tolerant germplasm. These results were consistent with grain filling being limited by assimilate supply from the heat-damaged photosynthetic apparatus, or alternatively, accelerated maturation in the grains that was correlated with leaf senescence responses merely due to common genetic control of senescence responses in the two organs. There was no evidence for a role of mobilized stem reserves (water soluble carbohydrates) in determining grain weight responses.

**Conclusions:**

Molecular markers for the 3B or 6B loci, or the facile measurement of chlorophyll loss over the heat treatment, could be used to assist identification of heat tolerant genotypes for breeding.

**Electronic supplementary material:**

The online version of this article (doi:10.1186/s12870-016-0784-6) contains supplementary material, which is available to authorized users.

## Background

Wheat is a temperate crop best adapted to cool growing conditions. However, in the Australian wheat belt and may other parts of the world, temperatures increase during the wheat growing cycle, exposing the crop to damaging heat waves (one to several days of +30 °C temperatures) during the sensitive reproductive development stages (booting through to grain filling) [1]. In addition to reducing yield, these events decrease the average grain size and increase the proportion of very small grains (screenings), downgrading the value of the harvested grain at delivery. Average annual wheat yield losses due to heat stress in Australia and the USA have been estimated at 10–15 % [1]. Furthermore, the problem is expected to worsen with climate change. For example, it is estimated that within 35 years, over half of the Indo-Gangetic Plains (in India and Pakistan) - currently producing 15 % of the world’s wheat in one of the most populous regions - will become re-classified as a heat-stressed growing environment [2].

Heat stress that occurs at around meiosis can cause floret sterility, with the sensitivity to this effect peaking about 10 d before anthesis [3]. Floret sterility leads to a reduction in grain number. Heat stress that occurs early in grain filling can reduce grain size [4]. These narrow windows of susceptibility for specific yield components, coupled with the sporadic and unpredictable nature of natural heat events and their frequent co-occurrence with drought stress, hampers efforts to breed for heat tolerance by direct selection. Greater scientific knowledge about traits associated with heat tolerance, and molecular markers for loci that affect those traits, could be useful for devising more effective selection methods.

A range of physiological and biochemical processes limit wheat yields under high temperature conditions and any of these could potentially represent the basis for genotypic variation in heat tolerance (reviewed by Cossani and Reynolds, 2012 [5]). Heat stress accelerates the loss of leaf chlorophyll, reducing photosynthetic capacity and supply of assimilate to the filling grains. Hence, the ability of some genotypes to maintain green area longer under stress (‘stay-green’) is considered an advantage [6]. Another source of assimilate is water soluble carbohydrate mobilized from the stems to the filling grains, particularly under stress conditions that limit current photosynthesis [7]. Vulnerability of the starch biosynthetic capacity of the grain itself may also be a critical factor, notably in relation to heat sensitivity of soluble starch synthase in the developing grain [8] and accelerated maturation of the grain by heat, triggered by stress signals such as ethylene [9]. Elevated temperatures increase evaporative demand, potentially causing moisture stress. Open stomata enabled by a favourable plant water status are also necessary for photosynthesis and also allow evaporative cooling of the plant tissues through transpiration. Lower canopy temperature has been found to correlate with yield performance in various heat/drought stressed environments [10].

Mapping of heat tolerance quantitative trait loci (QTL) is a pre-requisite for producing molecular markers suitable for heat tolerance breeding. QTL co-localization can also be a powerful way of identifying traits associated with heat-tolerance of yield components. These associated traits can give clues about underlying tolerance mechanisms and potentially provide complementary selection criteria for heat tolerance breeding. A number of researchers have mapped QTL for heat tolerance in wheat based on relative performance in late- versus timely-sown field experiments [10–15]. However the relevance of these QTL to heat shock events experienced in the normal production environment is uncertain due to the various other ways that late sowing alters plant performance [16]. While the growing environment in greenhouse/growth-chamber experiments also differs in several important ways to the field [17], at least such experiments allow a controlled and precisely timed heat treatment to be applied to one set of plants that otherwise experience the same growing conditions as their controls. Controlled environment screens therefore provide a practical approach for identifying heat tolerance QTL that can be subsequently tested for reproducibility in the field, e.g., by evaluating weather parameter x genotype interactions in multi-site and -location trials of near-isogenic lines.

There have only been a few studies to map QTL for heat tolerance of yield components and associated traits in wheat. Mason and colleagues detected tolerance QTL for yield components and architectural traits in one mapping population [18], and for yield components and organ temperature in another [19]. Two other studies focussed only on kernel weight [20] or traits relating to chlorophyll content dynamics [21].

In the current study we sought to expand the knowledge of heat tolerance QTL for yield components in wheat and their associations with heat-response and *per se* parameters relating to chlorophyll content and plant architecture, by applying greenhouse/chamber heat tolerance assays to a new doubled haploid mapping population made from a cross between the Australian varieties Drysdale and Waagan. The heat treatment was applied at 10 d after anthesis (DAA) to produce effects on final grain size.

## Results

### Comparison of experiments, trait and parents

Temperatures in the greenhouse where plants were grown before and after heat treatments are shown in an Additional file 1: Table S1. Temperature was constant and similar, except that in Experiment 2 there were 9 days over 30 °C at around anthesis and 13 days over 30 °C at around grain filling due to high outside temperatures. In the greenhouse, which was naturally lit, plants in Experiment 2 began growing under short days and matured under long days, whereas the converse occurred in Experiment 1.

**Fig. 1:**
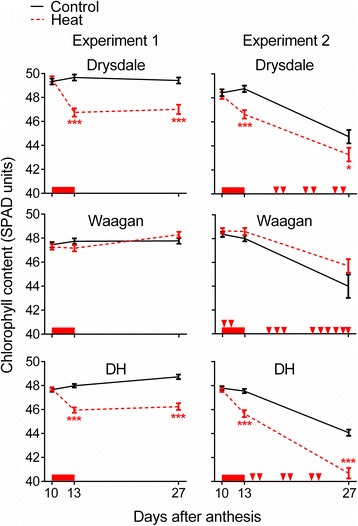
Time-courses of chlorophyll content (SPAD measurements) during and 2-weeks after the 3 d heat treatment. The red bar represents the period of heat treatment. The triangles for Experiment 2 indicate >30 °C days in the greenhouse. Error bars show SEM. * and *** indicate significant difference between control and heat-treated plants at *p* < 0.05, and *p* < 0.001, respectively

Means and standard error (SE) of all traits in the two parents and doubled haploids (DHs) across the two treatments and experiments are shown in an Additional file 2: Table S2. On average, plants in Experiment 2 took ~20 % longer to reach anthesis (days to anthesis, DTA), and were larger (~50–70 % more grains spike^−1^, GNS, had greater flag leaf length, FL and width, FW, ~40–60 % greater shoot weight, ShW, and had slightly greater plant height, PH). However, they took less time to senesce completely in the spike and flag leaf after anthesis (grain filling duration, GFD and period from anthesis to 95 % flag leaf senescence, FLSe, shortened by ~8–30 %). Despite this shorter post-anthesis green period and the greater number of hot days in the ‘control’ greenhouse in this experiment, the grains were ~20–40 % larger than in Experiment 1. Time course plots of flag leaf chlorophyll (Fig. 1) illustrate that during the period of measurement (10–27 DAA), control plants underwent senescence in Experiment 2 but not in Experiment 1.

Table 1 shows the % heat response (heat treated plants vs. control plants) of each trait in the two experiments, in the parents and DH mapping lines. As expected, the heat treatment did not significantly affect GNS, DTA or chlorophyll content at 10 days after anthesis (ChlC10DAA), as these were traits that were established prior to heat treatment. Significant heat effects included reduced grain size (grain weight spike^−1^, GWS, single-grain weight, SGW and harvest index, HI), reduced the time to reach complete senescence of the spike and flag leaf (GFD, FLSe and days to maturity, DTM) and accelerated flag leaf chlorophyll loss (chlorophyll content at 13 DAA, ChlC13DAA and at 27 DAA, ChlC27DAA, the area under the SPAD curve, AUSC, and chlorophyll loss rates during the treatment, ChlR13, and from directly before the treatment to 27 DAA, ChlR27). Grain weight responses (GWS, SGW and HI) tended to be greater in Experiment 2 than Experiment 1 both in percentage and absolute terms, while chlorophyll and senescence traits responded to heat similarly across experiments. In some cases, there was a significant reduction in ShW associated with the heat treatment.

**Table 1:** Trait responses. Responses are percent differences in heat treated plants relative to control plants, for the two parents and the means of the doubled haploids (DH)

	Experiment 1			Experiment 2		
Trait	Drysdale	Waagan	DH	Drysdale	Waagan	DH
DTA	0.54	0.77	0.07	0.15	−2.13	0.04
DTM	−2.84**	−1.93*	−4.36***	−3.71***	−3.53***	−3.08***
GFD	−5.98***	−5.85***	−8.66***	−8.31***	−6.17***	−7.3***
FLSe	−8.08***	−2.39	−12.52***	−6.4**	2.88	−3.88***
GWS	0.58	1.94	−4.58**	−11.08***	−10.65***	−10.93***
GNS	2.69	−0.58	−0.46	−2.8	−2.02	0.61
SGW	−2.07	2.49	−4.07***	−8.21***	−8.79***	−11.12***
ShW	2.03	4.14	−2.88*	−2.34	−7.17*	−1.55
PH	1.77	−0.01	−0.06	0.08	0.07	0.48
ChlC10DAA	0.41	−0.38	0.13	−0.47	0.6	−0.31
ChlC13DAA	−5.88***	−1.21	−4.25***	−4.37***	1.23	−3.97***
ChlC27DAA	−4.88***	1.09	−5.11***	−3.33*	3.89	−7.67***
AUSC	−4.91***	−0.19	−3.48***	−3.6**	3.71	−5.01***
ChlR13	−1.04***	−0.14	−0.7***	−0.63***	0.11	−0.58***
ChlR27	−0.1***	0.06	−0.05***	−0.03	0.18	−0.15***
FL	2.27	−0.29	0	0.82	3.72	0.41
FW	1.28	−0.65	−0.65	1.64	−1.59	0
HI	−0.57	−0.75	−0.9	−3.85***	−1.9**	−4***

*, **, and *** indicate significant difference between control and heat-treated plants at *p* < 0.05, *p* < 0.01, and *p* < 0.001, respectively

*DTA* days from sowing to anthesis, *DTM* days from sowing to maturity defined as 95 % spike senescence, *GFD* grain-filling duration defined as days from anthesis to 95 % spike senescence, *FLSe* days from anthesis to 95 % flag leaf senescence, *GWS* grain weight spike^−1^, *GNS* grain number spike^−1^, *SGW* single grain weight, *ShW* shoot dry weight, *PH* plant height, *ChlC10DAA* chlorophyll content 10 days after anthesis, i.e., just before heat treatment period, *ChlC13DAA* chlorophyll content 13 days after anthesis, i.e., just after heat treatment period, *AUSC* area under the SPAD curve made from measurements at 10, 13 and 27 days after anthesis, i.e., incorporates the period during-heat treatment and 2-weeks after, *ChlR13* rate of chlorophyll change between 10 and 13 days after anthesis, i.e., during the heat treatment period, *ChlR27* rate of chlorophyll change based on the linear regression of the measurements, at 10, 13 and 27 days after anthesis, *FL* flag leaf length, *FW* flag leaf width, *HI* harvest index

Traits are partitioned in the table based on their relationships to duration of development phases, yield components and biomass, chlorophyll content and stability, flag leaf dimensions and harvest index, respectively

Relative to Drysdale, Waagan took longer to reach anthesis (DTA), but took less time to senesce completely in spikes and flag leaves after anthesis (shorter GFD and FLSe), had more grains spike^−1^ (GNS) but smaller grains (SGW), had shorter PH and had shorter flag leaves (FL) (Additional file 2: Table S2). In Drysdale (and on average the DHs), flag leaf chlorophyll was reduced by heat during the 3 day treatment (ChlC13DAA; Table 1) but thereafter the plants recovered to resume chlorophyll loss rates similar to those of controls (Fig. 1). By contrast, the tolerant parent Waagan showed no significant effect of heat on chlorophyll loss measured up to 27 DAA (ChlC13DAA or ChlC27DAA) or on the time taken for flag leaves to completely senesce (FLSe) (Table 1; Fig. 1). Significant heat responses of grain weight (GWS or SGW) were observed in Waagan and Drysdale, but only in Experiment 2, and the responses were similar between the varieties (~11 % for GWS and 8.5 % for SGW) (Table 1).

### Trait heritabilities

Trait heritabilities (H^2^) in the DH lines are shown in an Additional file 3: Table S3. These were large for plant height, shoot weight and yield components, owing to segregation of the *Rht-B1* and *Rht-D1* semi-dwarfing genes. Heritability of grain size (SGW) was high under control conditions (~0.8) and did not increase under heat. By contrast, heritability of chlorophyll and senescence related traits increased markedly under heat, which at least partially reflected the presence of segregating genes influencing heat-induced senescence (see next sections).

### Trait correlations

Heat responses were defined using the heat susceptibility index (HSI) of Fischer and Maurer [22] (see Methods), which describes the performance of the genotype under control conditions relative to heat, normalized for the stress intensity of the experiment. Correlations between trait potentials (under control conditions) and trait HSIs are represented in an Additional file 4: Table S4.

In Experiment 1, which was sown in early autumn, earlier flowering genotypes tended to have greater grain weight stability under heat (positive correlation between DTA in control and HSI of SGW and GWS) whereas in Experiment 2 sown in mid-winter, there were no significant correlations with flowering time.

Larger plant size (greater GWS, GNS, SGW, ShW and plant height, PH) tended to be positively correlated with stability of chlorophyll traits (FLSe, ChlC27DAA, AUSC, ChlR27) (i.e., negative correlation with HSI) and grain size traits (GWS or SGW) in Experiment 1, whereas the trend was the opposite in Experiment 2.

In both experiments, genotypes with more chlorophyll *per se*, slower senescence and a longer period of post-anthesis flag leaf greenness under control conditions also tended to maintain chlorophyll, grain weight and shoot weight better under heat (negative correlation between FLSe, ChlC10AA, ChlC13DAA, ChlC27DAA, AUSC, ChlR13 and ChlR27 under control and HSIs of SGW, GWS, ShW and most chlorophyll traits), particularly in Experiment 2. Exceptions to this trend were the traits describing the duration of post-anthesis greenness in the spikes and flag leaves (GFD and FLSe, respectively) in Experiment 1, for which there were positive correlations between control values and HSIs (Additional file 4: Table S4).

Overall, these correlations indicate that earlier flowering, greater greenness (*per se* and heat stability) and heat stability of shoot weight were associated with the ability to maintain grain weight under heat.

### Segregation of dwarfing and flowering time genes

The QTL analysis and diagnostic markers showed that the only major phenology loci segregating in the Drysdale × Waagan doubled haploid population were *Rht-B1* and *Rht-D1* for plant height (PH). Drysdale carried the wild-type (tall) allele at *Rht-B1* and dwarfing allele at *Rht-D1*, and vice versa for Waagan. The strongest QTL for days to anthesis (DTA) had an additive effect of only 1.6 d (Additional file 5: Table S5 and Additional file 6: Table S6), and the population was uniform for diagnostic marker-polymorphisms at *Vrn-A1* (winter allele), *Vrn-B1* (spring allele), *Vrn-D1* (spring allele) and *Ppd-D1* (photoperiod insensitive allele). In the *Rht8* region on chromosome 2D, there were no QTL for height. Consistent with non-segregation for *Rht8*, all DH gave a *gwm261* microsatellite marker fragment of the same size (~165 bp, similar to the cv. Chara control; not shown).

The three minor flowering time QTL were on linkage groups 2B2, 4B, and 7B. The population segregated for the (non-diagnostic) *Ppd-B1* marker at the 74 cM location on linkage group 2B1, but the minor flowering time effect (QTL6) mapped at position 5 cM. Hence this was not a *Ppd-B1* effect.

### The molecular marker map

The linkage map made from the Drysdale × Waagan DH population is represented in Additional files, using the total mapped marker set (Additional file 7: Table S7) or non-redundant marker set (Additional file 8: Figure S1). Its features are summarized in an Additional file (Additional file 9: Table S8). It consisted of 551 genetically non-redundant marker loci spanning a total of 2,447 cM, at an average marker spacing of 4.4 cM (not counting 16 gaps between linkage groups within chromosomes).

### Heat tolerance QTL

There was a total of 29 QTL regions defined (numbered QTL1-QTL29) (Additional file 5: Table S5; Additional file 10: Figure S2). Of these, ten showed significant HSI QTL (tolerance) effects. Only two of these (QTL11 on chromosome 3B and QTL27 on chromosome 6B) showed HSI effects for grain weight (SGW or GWS) and these are summarized in Table 2. For simplicity, only the two SGW HSI effects were given formal QTL names for future reference (*QHsgw.aww-3B* and *QHsgw.aww-6B*).

**Table 2:** QTL effects locating to QTL11 and QTL27, the only loci in the Drysdale × Waagan population that showed heat-tolerance effects for single grain weight

QTL	Trait	Condition	Expt.	Positive allele	Test statistic	R^2^	Additive effect
					-Log_10_(p)		
QTL11	ChlC10DAA	Pre-heat	1,2	W	11, 7.4	18, 17	0.71, 0.65
QTL11	ChlC13DAA	Control	1,2	W	7.7, 8.9	15, 19	0.59, 0.67
QTL11	ChlC27DAA	Control	1,2	W	8.6, 12	17, 23	0.66, 0.68
QTL11	AUSC	Control	1,2	W	7.5, 11	15, 20	10.8, 11
QTL11	HI	Control	2	D	8.7	12	0.94
QTL11	GFD	Heat	1	W	6.4	13	0.76
QTL11	FLSe	Heat	1	W	5.5	14	2.06
QTL11	GWS	Heat	1	W	7.1	11	0.11
QTL11	SGW	Heat	1	W	7.0	12	1.65
QTL11	ShW	Heat	1,2	W	18, 5.8	14, 3.5	0.14, 0.1
QTL11	ChlC13DAA	Heat	1,2	W	26, 16	42, 34	2.01, 1.95
QTL11	ChlC27DAA	Heat	1,2	W	36, 8	54, 20	2.49, 2.55
QTL11	AUSC	Heat	1,2	W	33, 13	49, 28	37, 34.3
QTL11	ChlR13	Heat	1,2	W	20, 13	40, 27	0.37, 0.37
QTL11	ChlR27	Heat	1	W	30.0	50	0.07
QTL11	GFD	HSI	1	D	4.4	10	0.12
QTL11	FLSe	HSI	1	D	6.7	14	0.27
QTL11	GWS	HSI	1,2	D	8.8, 5.9	22, 15	1.16, 0.38
QTL11	SGW	HSI	1,2	D	8.1, 4.7	20, 11	0.92, 0.16
QTL11	ShW	HSI	1	D	9.3	23	1.62
QTL11	AUSC	HSI	1,2	D	21, 7.3	38, 18	0.91, 0.67
QTL11	ChlC13DAA	HSI	1,2	D	17, 10	36, 24	0.67, 0.81
QTL11	ChlC27DAA	HSI	1,2	D	21, 5.3	39, 13	1.28, 0.62
QTL11	ChlR13	HSI	1,2	D	16, 13	40, 27	0.55, 0.8
QTL11	ChlR27	HSI	2	D	9.3	19	0.40
QTL11	HI	HSI	2	D	4.1	10	0.29
QTL27	AUSC	Heat	2	D	3.7	9.1	19.53
QTL27	ChlR13	Heat	2	D	4.8	8.9	0.21
QTL27	SGW	HSI	2	W	3.8	12	0.17
QTL27	ChlR13	HSI	2	W	4.8	8.9	0.46

Where corresponding QTL effects were identified in both experiments, the positive allele was always the same; for other attributes, values for Expt. 1 and 2 are shown separated by a comma

Positive allele: *D* Drysdale, *W* Waagan, Positive allele for Heat Susceptibility Index (HSI) means associated with intolerance

Additive effect always refers to the effect of the positive allele

*DTA* days from sowing to anthesis, *DTM* days from sowing to maturity defined as 95 % spike senescence, *GFD* grain-filling duration defined as days from anthesis to 95 % spike senescence, *FLSe* days from anthesis to 95 % flag leaf senescence, *GWS* grain weight spike^−1^ (g), *GNS* grain number spike^−1^, *SGW* single grain weight (mg), *ShW* shoot dry weight (g), *PH* plant height (cm), *ChlC10DAA* chlorophyll content 10 days after anthesis, i.e., just before heat treatment period (SPAD units), *ChlC13DAA* chlorophyll content 13 days after anthesis, i.e., just after heat treatment period (SPAD units), *AUSC* area under the SPAD curve made from measurements at 10, 13 and 27 days after anthesis, i.e., incorporates the period during-heat treatment and 2-weeks after, *ChlR13* rate of chlorophyll change between 10 and 13 days after anthesis, i.e., during the heat treatment period (SPAD units day^−1^), *ChlR27* rate of chlorophyll change based on the linear regression of the measurements, at 10, 13 and 27 days after anthesis (SPAD units day^−1^), *FL* flag leaf length (cm), *FW* flag leaf width (cm), *HI* harvest index (%)

### QTL11 on chromosome 3B

The strongest QTL for HSI of grain weight (SGW and GWS) (QTL11) was located distally on the tip of the short arm of chromosome 3B. Its attributes are shown in Table 2. It was detected in both experiments and accounted for 11 to 22 % of the variance, with the Waagan allele conferring grain weight stability (lower HSI). On average, the Waagan allele reduced heat induced losses of SGW by 2.5 mg and 1.7 mg over the Drysdale allele in Experiments 1 and 2, respectively, where the average reduction due to the heat treatment in the DHs was 1.2 mg and 5.4 mg, respectively. The strongest HSI QTL for each of the chlorophyll related traits (accounting for ~13 and 40 % of the variance) were also observed at this QTL position (with the exception of FLSe which showed an HSI QTL effect of similar magnitude at QTL18 in Expt. 1) (see Additional file 6: Table S6). For these HSI effects, the Waagan allele also favoured greater chlorophyll stability, in terms of absolute content after heat treatment (ChlC13DAA, ChlC27DAA and AUSC), senescence rate (ChlR13 and ChlR27) and the time taken for the flag leaf to senesce completely after anthesis (FLSe). In other words, the effect of this locus on heat tolerance for grain size was associated with stay-green. In Experiment 1, the Waagan allele at the locus stabilized shoot weight (the only ShW HSI QTL detected) and grain filling duration (GFD) under heat (one of three such QTL). These effects on ShW and GFD were further indications of the ability of the Waagan QTL11 allele to slow senescence in plants exposed to post-anthesis heat.

As shown by the data in Table 2, the HSI QTL effects at QTL11 were mainly/solely derived from genetic effects expressed under heat conditions rather than control conditions, i.e., this locus gave significant QTL effects under heat conditions but not under control conditions, for traits related to grain size (SGW, GWS and GFD), and senescence rate (ChlR13, ChlR27, FLSe) and for shoot weight (ShW). For flag leaf chlorophyll content, both before the heat treatment period (ChlC10DAA) and after (ChlC13DAA, ChlC27DAA and AUSC), the Waagan allele of QTL11 also conferred higher values *per se* in control plants, although this effect increased under heat. Under control conditions, the Waagan allele also favoured lower HI, although no QTL effects were detected at this locus for the components of HI (GWS or ShW).

### QTL27 on chromosome 6B

The only other locus to show a tolerance effect for grain weight was QTL27 on chromosome 6B (SGW effect only; Table 2). The tolerance allele from Drysdale was associated with a reduced rate of heat-induced chlorophyll loss during the heat treatment (ChlR13; same association as at QTL11), as well as a less negative ChlR13 and greater AUSC *per se* under heat. These effects were weaker and less consistent than those detected at QTL11, explaining only 8.9 to 12 % of the variation for these traits, and were detected only in the winter-sown experiment (Experiment 2). On average, the Drysdale allele reduced heat-induced SGW loss by 2.1 mg over the Waagan allele (Experiment 2, where the average reduction due to heat in the DH lines was 5.4 mg).

### HSI loci for traits besides grain weight

Six other loci showed HSI effects and these effects related to senescence traits (GFD, ChlR27, ChlC27DAA, AUSC and FLSe). The loci were on chromosomes 1A (QTL2), 4A (two, QTL13 and QTL 15), 4B (QTL18), 5A (QTL21) and 7B (QTL29). HSI effects on GFD and FLSe at QTL15, QTL18 and QTL21 were comparable in magnitude to those controlled by the major tolerance locus on 3B (QTL11), while the other effects at these loci were weaker than that of QTL11 (Additional file 6: Table S6).

The ChlR27 tolerance (Waagan) allele at QTL2 was associated with lower chlorophyll content pre-heat (ChlC10DAA), which was the opposite relationship to the one observed at QTL11. However it did confer less negative ChlR27 (slower chlorophyll loss rate) under control conditions, consistent with the other observations linking slower senesce under control to stay-green under heat.

A variety of trait behaviors were observed at the remaining HSI loci. QTL18 and QTL29, which were the two strongest flowering time loci segregating in the population (with effects of ~1.5 d), had rapid flowering alleles associated with heat tolerance of FLSe (and at QTL18, heat tolerance of GFD and ChlR27). However, under control conditions, the rapid flowering allele was associated with higher GFD and FLSe at QTL18 but lower GFD and FLSe at QTL29. QTL15 was one of four minor height loci detected (behind *Rht-B1* and *Rht-D1*). The tall allele was associated with heat sensitive GFD. The GFD tolerance allele at QTL21 was associated with longer GFD and greater SGW in control plants. Sensitivity to heat induced chlorophyll loss at QTL13 was associated with shorter GFD in control plants.

### Other loci affecting grain weight under heat stress

Three QTL relating to grain weight (SGW or GWS) were detected under only heat conditions but didn’t translate to significant grain weight HSI effects. These were located on chromosomes 1B (QTL3), 4A (QTL14) and 6B (QTL26). The large-grain alleles at QTL14 and QTL26 were also associated with greater shoot weight under heat, and the latter also with greater grain number per spike under heat.

Five other loci showed SGW effects under both heat and control conditions but no HSI effect for SGW. These were on chromosomes 2D (QTL9), 4B (QTL17 = *Rht-B1*), 4D (QTL19 = *Rht-D1*), 5B (QTL23) and 6A (QTL25).

### Relationship of QTL11 to previously documented QTL in wheat

**Fig. 2:**
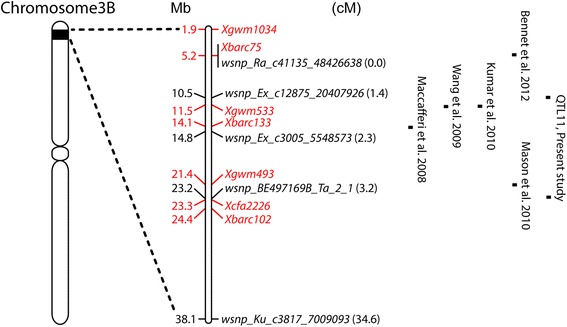
Previously described QTL in the vicinity of the QTL11 heat tolerance locus. QTL positions were compared based on positions of markers from the current study (*black*, with cM positions shown in brackets) and previous studies (*red*) in the reference wheat chromosome 3B sequence. Numbers to the left of the magnified chromosome segment indicate Mb distance from the top of the chromosome. QTL are marked by peak (or nearest placed) marker positions (for current study, for grain weight stability QTL). Other published QTL effects were: Grain yield and plant height in stressed and other environments in a durum wheat RIL population (Kofa × Svevo; markers *Xbarc133*/*Xgwm493*) [26]; heat tolerance index for grain number spike^−1^ for a brief heat stress applied at 10 days after anthesis in a growth chamber, and flag leaf length before heat treatment, in a spring × winter wheat cross (Halberd × Cutter; markers *Xbarc75*/*Xgwm493*) [18]; stay-green visually scored under high temperature field conditions in a bread wheat RIL population (Chirya3 × Sonalika; marker *Xgwm533*) [24]; maximum grain filling rate, grain filling duration, thousand grain weight, and flowering time under field conditions in a winter bread wheat RIL population (HSM × Y8679; marker *Xgwm533*) [25]; chlorophyll content under drought/heat or irrigated conditions in Mexico in a spring bread wheat DH population (RAC875 × Kukri; marker *Xbarc75*) [23]

Markers most commonly associated with peaks of QTL effects at the QTL11 locus (*wsnp_Ra_c41135_48426638* at 0 cM to *wsnp_BE497169B_Ta_2_1* at 3.5 cM) delimited an 18 Mb region on the wheat chromosome 3B reference sequence, representing ~2.3 % of total physical length of the 774 Mb chromosome. Other previously reported QTL on 3BS were able to be located in this vicinity, based on sequence matches of closely linked markers to this part of the 3B reference sequence (Fig. 2).

There were some differences as well as similarities between the effects of QTL11 and the other previously reported QTL. As for QTL11, the QTL of Bennett et al. [23] and Kumar et al. [24] affected the content and stability of leaf chlorophyll while the QTL of Wang et al. [25] influenced single grain weight and grain growth. The QTL of Maccaferri et al. [26] influenced yield in the field. Differences in the phenotype of QTL11 relative to the other QTL include a plant height effect at the durum locus, a flowering time effect at the Wang et al. [25] locus, a flag leaf length effect at the locus of Mason et al. [18], and the lack of a significant grain size effect under heat/drought stress conditions at the loci of Bennett et al. [23] and Mason et al. [18]. These comparisons suggest that variation for QTL11 may be present in other germplasm and express a yield and/or grain size effect under field conditions.

## Discussion

This greenhouse-chamber study identified two QTL influencing response of final grain size to a brief severe heat stress treatment applied at early grain filling, with a locus on 3BS being the strongest and most reproducible. Single grain weight (SGW) and its response to heat represents the integration of many processes. Therefore, we measured a range of physiological and developmental traits to gain insights into factors driving heat responsiveness to grain weight and the basis of the tolerance mechanisms controlled by the QTL.

### Relationships of SGW heat tolerance effects to photosynthetic capacity - flag leaf chlorophyll and flag leaf dimensions

The heat treatment reduced chlorophyll in the flag leaves, mainly during the 3 d heat treatment period (Fig. 1). Consistent with the idea that this chlorophyll loss affected grain weight, the major QTL conditioning grain weight maintenance under heat (QTL11) also showed the strongest QTL effects for chlorophyll response parameters, with the Waagan allele for stable SGW contributing to retention of flag leaf chlorophyll under heat. QTL11 accounted for 54 % of the phenotypic variation for ChlC27DAA (in Experiment 1). On average, DH lines carrying the Drysdale allele lost 5.0 more SPAD units by 27 DAA than those carrying the Waagan allele (out of an average starting value of 47 SPAD units), compared to an average of 2.5 SPAD units lost across all DHs.

Generally, QTL11 also had the greatest effect on chlorophyll content *per se* traits in control plants. Four other loci also affected chlorophyll content *per se* (QTL2, QTL5, QTL8 and QTL20). These four loci also influenced tolerance to heat-induced chlorophyll loss, with the high chlorophyll *per se* alleles favouring chlorophyll stability. However, none of these loci produced significant SGW HSI effects. The weaker SGW tolerance locus QTL27 also showed no effect on chlorophyll *per se* in control plants. QTL27 was, however, the only locus other than QTL11 to show a significant QTL effect for the rate of decline in chlorophyll during the heat treatment period (ChlR13 trait) in the heat treated plants and for ChlR13 heat responsiveness (ChlR13-HSI). Therefore, among the chlorophyll traits, ChlR13 (and HSI of ChlR13) was the most consistent indicator of SGW heat tolerance QTL.

Why high chlorophyll content *per se* under control conditions should be related to chlorophyll heat-resilience is unclear, but might involve a more active state of chlorophyll synthesis capable of buffering against heat induced chlorophyll losses. One possibility is that low-chlorophyll *per se* genetic effects derived from an earlier onset of leaf senescence (relative to anthesis), and therefore ‘priming’ for more rapid heat-induced chlorophyll losses. Unfortunately, the presence of multiple hot (>30-degree) days during grain filling in the control greenhouse in Experiment 2 prevented us from estimating the senescence status of the plants in that experiment prior to heat treatment (Fig. 1). 

The coupling of grain weight and flag leaf chlorophyll responses at QTL11 and QTL27 could imply that heat-induced chlorophyll loss in susceptible genotypes reduced photosynthate supply to a point that it became limiting to grain filling. Photosynthesis in flag leaves (and spikes) provides a major source of assimilates for grain filling in wheat [27]. Under optimum growth conditions, grain filling is most commonly limited by sink strength, while a shift towards source limitation tends to occur under stress conditions such as drought which reduces green leaf area and/or photosynthetic efficiency [28]. Plants in Experiment 1 which were sown ’off season’ set and filled grain during the low light conditions of winter; however, they suffered less from heat-induced grain weight loss than those in Experiment 2 (both in percentage and absolute terms), probably owing partly to the fact they had fewer grains per spike (smaller sink size). Hence, it is uncertain if the ~5 % chlorophyll loss caused by the heat treatment was sufficient to cause source limitation in either experiment. Alternatively, curtailing of starch synthetic capacity in the grain through senescence responses within the grain itself may have been responsible for the grain weight losses. i.e., acceleration of senescence in the grains and flag leaves by heat may have been synchronized via common genetic control, rather than arising by a direct cause-effect relationship.

Another factor that could potentially influence photosynthetic capacity was flag leaf dimensions (FW and FL). However, QTL11 and QTL27 loci for heat tolerance of SGW had no detectable effect on these variables. FW and FL QTL effects were detected at other genomic locations, although these were minor (additive effects up to 4.5 mm for length and 0.6 mm for width) (Additional file 6: Table S6). Hence, we found no evidence that flag leaf dimensions (and by inference, area) impacted SGW heat tolerance, similar to the findings of Mason et al. [18].

### Relationships of SGW heat tolerance effects to the duration of grain filling and flag-leaf senescence

Short heat events during grain filling reduce final grain weight in wheat mainly by affecting grain filling duration rather than grain filling rate [4]. In the present study, the time between anthesis and 95 % senescence of the spikes on the main tiller was used as a measure of grain filling duration (GFD). Like GFD, the flag leaf senescence trait (FLSe) (time from anthesis to 95 % flag leaf senescence) relates to how long the top of the primary tillers remained green after anthesis, and these two traits were positively and closely correlated (Pearsons’ *r* = 0.66 to 0.71 under control and heat, respectively; *p* < 0.001). Spike photosynthesis can contribute a high proportion of the grain yield (e.g., 12–42 %; [29]), and this fraction tends to increase further under stress conditions such as drought [28]. Hence the GFD trait also had potential to relate to photoassimilate supply to the filling grains.

The main heat tolerance locus for grain weight (QTL11) expressed significant GFD and FLSe HSI effects, with the Waagan allele conferring heat stability of all three traits. However, the minor grain weight heat tolerance locus (QTL27) showed no significant GFD or FLSe HSI effects. HSI effects were observed for GFD at QTL15, QTL18 and QTL21 and for FLSe at QTL18 and QTL29. These HSI effects for GFD and FLSe were similar in magnitude to those of the SGW heat-tolerance locus QTL11 but these loci did not themselves significantly influence HSI of SGW.

QTL18 and QTL29 differed from QTL11 in that they also influenced time from sowing to anthesis (DTA). In both cases the late flowering allele made GFD and FLSe more responsive to heat and also resulted in shorter GFD and FLSe *per se* in control plants. Such a negative correlation between the duration of pre- and post- anthesis development has been reported before in both wheat and barley [30, 31], suggesting there is a general physiological link between time to flowering and the duration of post anthesis development in cereals. Dwarfing alleles at *Rht-B1* and *Rht-D1* loci lengthened GFD and FLSe *per se* in control conditions, but they gave no significant HSI effects for GFD or FLSe.

In summary, truncation of grain filling and/or responsiveness of green period duration in flag leaves or spikes were not consistent or strong features of SGW heat tolerance loci. However, this is based on the assumption that visual scoring of spike senescence provided an accurate proxy for GFD.

### Relationships of SGW heat tolerance effects to shoot mass

QTL11 was the only locus to show a significant effect on HSI of shoot dry weight at maturity (ShW), with the Waagan allele conditioning heat stability of SGW and ShW (as well as flag leaf chlorophyll). A plausible scenario is that the accelerated heat-induced chlorophyll loss associated with the Drysdale allele reduced the carbon fixing capacity of the plant, which in turn constrained both the ability to maintain/add dry matter in the shoots and possibly also the grain. Two other loci (QTL14 and QTL26) significantly affected both SGW and ShW only under heat (with the same allele conferring stability of both SGW and ShW at each locus), providing further evidence that heat stability of ShW and SGW was physiologically linked.

Conversely, the data did not support a hypothesis in which mobilization of water soluble carbohydrate (WSC) reserves from the stems contributed to grain weight stability under heat. This is because such a tolerance mechanism would be associated with a greater rather than smaller loss of ShW dry mass under heat. Tall alleles of the *Rht-B1* and *Rht-D1* loci increase absolute quantities of stem reserve (e.g., by 35 to 39 %, [32]) due to their effects on stem length. The fact that these loci had no measurable effect on grain weight maintenance under heat also argues against a contribution of stem WSC to grain weight stability in these conditions.

### Implications for breeding

This study detected several QTL with potential for use in marker assisted breeding. However, to determine whether they are worthy of use, the yield and/or grain size benefits of these QTL need to be verified in heat affected field trials (e.g., using near-isogenic lines). QTL11 showed the most promise, as it had the largest SGW-stabilizing effect under heat stress and this was expressed both in the mid-winter and early-autumn sown experiments. Previously described QTL in the vicinity (Fig. 2) suggest that QTL11 may vary within other germplasm and express yield and grain weight effects in the field. The other SGW heat tolerance locus (QTL27) had weaker effects and was detected in only one experiment (albeit the ‘in-season’ experiment) and hence seems less promising.

Together with loss of shoot weight during heat, the rate of chlorophyll loss in flag leaves during the brief heat treatment (ChlR13 trait) was the most diagnostic feature of SGW heat tolerance loci, and hence this trait showed promise as an indicator for SGW tolerance that might be useful in heat tolerance screening. Plants could be heat treated at early grain filling using either a growth chamber or by utilizing natural heat waves in the field. Its measurement would require no non-stressed controls and just two SPAD measurements - one directly before the heat treatment (or forecasted heat wave in the field) and one directly after.

Three QTL had grain size effects detectable only under heat (QTL3, QTL12 and QTL14) and these could be selected to provide an advantage under heat stressed environments. Another three loci (not counting *Rht-B1* and *Rht-D1*; QTL9, QTL23 and QTL25) affected grain size under both control and heat conditions and could therefore be selected to provide a grain weigh advantage under all conditions.

### Variety heterogeneity and linkage map construction

While varieties are required to be ‘distinct, uniform and stable’ for plant variety protection, a level of heterogeneity is tolerated, including at the marker level. Consequently, many released varieties (unless made by the DH technique) are heterogeneous for some genomic regions (e.g., as documented for glutenin loci) [33].

As described in Methods, heterogeneity in the parent varieties of the Drysdale × Waagan DH mapping population resulted in blocks of markers that segregated in some but not all ‘sub-populations’ (derived from different F_1_ plants). In total, there were 47 such blocks, spanning a total of 368 cM, or 15 % of the total genetic length of the genome. Prior to linkage map construction, we converted the marker scores in these blocks to ‘missing data’ to avoid mapping errors caused by spurious associations among markers.

Blocks affected by parent heterogeneity were defined as those that were non-segregating in 2 to 12 of 13 sub-populations. We expect that this approach was not fool proof, since some such blocks may have been missed (among those non-segregating in 1 sub-population) or incorrectly defined (among those non-segregating in a low number of sub-populations). These blocks could have been precisely identified if the parental plants used in crossing had been genotyped. Despite the absence of this information, there was good alignment of our map to a consensus map of the 9,000 SNP array [34], indicating that our map was largely accurate. Hence, our data processing approach allowed us to avoid most of the potential mapping errors due to parent variety heterogeneity. Fortunately, use of a high-density marker array and the availability of a reliable consensus map in this case allowed this approach to be applied.

## Conclusions

Two QTL were detected which influenced the response of grain weight to a brief heat stress applied at early grain filling in a growth chamber, QTL11 (*QHsgw.aww-3B*) and QTL27 (*QHsgw.aww-6B*), with the former having the strongest and most reproducible effect. Among the other measured traits, heat-induced losses in final shoot dry weight and increases in the rate of flag leaf chlorophyll loss during the heat treatment were the best predictors of loci affecting grain weight response, with alleles limiting grain weight loss also restricting loss of shoot dry mass and chlorophyll. Rate of chlorophyll loss during the heat treatment was identified as a trait warranting investigation as a potentially rapid genotype-screening tool to predict grain weight responses to heat shock events experienced in the field or imposed using chambers. Further work is required to establish whether the associations of chlorophyll, shoot weight and grain weight originate from source limitation to grain filling, or merely common genetic control of senescence in the leaves and grains. With validation, markers for QTL11 and QTL27 might prove useful in marker-assisted breeding of heat-tolerant wheat cultivars.

## Methods

### Plant material

This study used a Drysdale × Waagan F_1_-derived DH population and single-plant selections of the parental varieties to study the inheritance of heat tolerance in wheat. These varieties had been shown in our preliminary studies to contrast for grain weight and chlorophyll responses to heat.

Drysdale (Hartog*3/Quarrion) was released by GrainGene (AWB Limited, GRDC, Syngenta and CSIRO) in 2002 and was the first variety to be bred for increased water use efficiency by selecting the carbon isotope discrimination trait [35, 36]. It is best adapted to low/medium rainfall areas of Southern New South Wales and has also performed well in Victoria and South Australia. Waagan (Janz/24IBWSN-244; 24IBWSN-244 being a CIMMYT line) was released by the NSW Department of Primary Industries in 2007. From 2008 to 2012, Waagan was one of the highest yielding varieties in New South Wales, particularly in the north of the state [37, 38].

Seed of the parents were initially obtained from the NSW-DPI collection. Thirteen F_1_ plants were used to produce 184 DH lines using the maize pollination technique at the Plant Breeding Institute (Cobbitty, University of Sydney), with 5 to 31 DH being produced from each F_1_. The six Drysdale selections were derived from the same (female) parent plants that were used in crossing, while the 10 Waagan selections were made from randomly-selected plants that had been grown from the same seed packet as the (male) parent plants.

All DH lines and single-plant selections were genotyped for *Vrn*, *Ppd* and *Rht* markers (later section) and phenotyped, while the parent varieties were each reduced to two single-plant selections for scoring with the SNP array. SNP analysis showed that the 184 DH only represented 144 unique lines, as there were a number of lines with identical or highly similar marker genotypes. The latter were treated as unintentional replicates in deriving predicted trait means.

### Plant growth, heat stress and data collection

Heat stress assays were based on procedures used by others [39, 40]. Plants were grown one to a pot (8 × 8 cm, 18 cm depth) initially in a naturally-lit greenhouse in Adelaide set at 24/18 °C day/night, using the same soil, watering, fertilizer and temperature-control conditions as Maphosa et al. [41]. Experiment 1 was sown on 16^th^ of March 2012 (early-autumn) and Experiment 2 on 21^st^ of July (mid-winter) 2012. As in other heat tolerance studies [42, 43], plants were kept pruned back to the single main tiller to aid light interception and management. Anthesis date was recorded for each plant, and at 10 days after their respective anthesis dates half of the plants were moved individually into a growth chamber for a 3 d heat treatment of 37/27 °C day/night, before being returned to the greenhouse to reach maturity. The chamber and settings for the heat treatment were the same as used by Maphosa et al. [41].

Although plants were placed in shallow (~2 cm) trays of water to minimize drought stress during the heat treatment they became partially dehydrated in the shoots during the day-cycle due to the high evaporative demand, appearing mildly wilted and reaching leaf water potentials of −11 to −15 Bar, as determined using a Scholander bomb. We attempted to quantify transpiration rates in the plants but the infrared-camera malfunctioned under the high temperature and humidity conditions of the chamber.

Plants were evaluated for traits: Days from sowing to anthesis defined as when exerted anthers first became visible (DTA); days from sowing to maturity defined as 95 % spike senescence visually scored (DTM); grain filling duration defined as DTM minus DTA (GFD); days from anthesis to 95 % flag leaf senescence visually scored (FLSe); flag leaf width at the widest point (FW) and length of the blade (FL) measured at 10 days after anthesis; relative chlorophyll content of the flag leaf measured using a portable SPAD meter (SPAD-502; Minolta Co. Ltd., Japan) at 10, 13 and 27 days after anthesis, corresponding to directly before and after treatment and at 14 days after treatment, respectively (ChlC10DAA, ChlC13DAA and ChlC27DAA); the area under the curve of the three SPAD readings (AUSC) calculated with the formula:

$$ AUSC={\displaystyle {\sum}_{i=1}^{i-1}\left[\left(\frac{X_i+{X}_{\left(i+1\right)}}{2}\right)\right.}\times \left({t}_{\left(i+1\right)}-{t}_i\right), $$

where X_i_ is the relative chlorophyll content (SPAD units) on the i^th^ date, t_i_ is the date on which the chlorophyll content was measured, and n is the number of dates on which chlorophyll content was recorded; the linear rate of chlorophyll change between ChlC10DAA and ChlC13DAA representing the change over the heat-treatment time (ChlR13); the linear rate of chlorophyll change between 10 and 27 days after anthesis calculated from the linear regression of all three SPAD readings (ChlR27); at maturity: plant height measured from the soil surface to the tip of the spike of the primary tiller excluding awns (PH); dry weight of the primary tiller from the soil surface to bottom of spike after oven drying shoot at 85 °C for 3 d (ShW); grain weight per spike measured after grain weight stabilized at room temperature for ~4 weeks post-harvest (GWS); grain number per spike (GNS); single grain weight (SGW) calculated as GWS/GNS; harvest index (HI, %) calculated as (GWS/(GWS + ShW)) × 100. The heat susceptibility index [Fischer, 1978 #2695] was calculated for all traits using the formula:

$$ HSI=\left(1-{Y}_{Heat}/{Y}_{Control}\right)/\left(1-{X}_{Heat}/{X}_{Control}\right), $$

where *Y*_*Heat*_ and *Y*_*Control*_ are the means for each genotype under heat and control environments and *X*_*Heat*_ and *X*_*Control*_ are means of all lines under heat-treatment and control conditions, respectively.

### Experimental design and statistical analysis

Each experiment used a split-plot design with genotype (parents and DH lines) and temperature treatments (control vs. heat) as main plots and subplots respectively. The genotypes were assigned to main plots using a randomized block design and, for any given genotype, the control plant and the plant assigned to the heat treatment were neighbours within each main plot. Each DH was replicated twice and parent varieties were replicated 6 to 8 times.

Each trait within an experiment was analysed separately using a linear mixed model that accounted for genetic and non-genetic sources of variation. For the vector of trait observations, *y* = (*y*_1_, …, *y*_*n*_) the linear mixed model was defined as:

$$ y=X\tau +Zu+{Z}_{\mathit{\mathsf{g}}}\mathit{\mathsf{g}}+e $$

where *τ*, is a vector of fixed effects and *u* and *g* are vectors of non-genetic and genetic random effects, respectively. *X, Z* and *Z*_*g*_ are design matrices which associate the trait observation with the appropriate combination of fixed and random effects. The genetic effects were assumed to have distribution $g \sim N\ \left(0,\ {\Sigma}_{\mathit{\mathsf{g}}}\otimes {I}_{\mathit{\mathsf{g}}}\right)$ where Σ_*g*_ is a 2 × 2 matrix with diagonal elements $\left({\delta}_{{\mathit{\mathsf{g}}}_c}^2,{\delta}_{{\mathit{\mathsf{g}}}_h}^2\right)$ representing the genetic variance for the control and heat treatments and *I*_*g*_ is the identity matrix. The residual error was assumed to be distributed as *e* ~ *N* (0, *δ*^2^*R*(*ρ*_*r*_ , *ρ*_*c*_)) where *δ*^2^ is the residual variance and *R*(*ρ*_*r*_ , *ρ*_*c*_) is a correlation matrix containing a separable AR1 × AR1 autoregressive process with parameters *ρ*_*r*_ and *ρ*_*c*_ representing the correlation along the rows and columns of the experimental layout. For each of the traits within each experiment a generalized heritability (*H*^2^), which is an estimate of the broad-sense heritability, developed by Cullis et al. [44] and Oakey et al. [45] was calculated for each treatment using:

$$ {H}^2=1-\frac{E}{2{\delta}_{\mathit{\mathsf{g}}}^2} $$

where *E* is the average pairwise prediction error variance of the best linear unbiased predictors (BLUPs) and ${\delta}_{\mathit{\mathsf{g}}}^2$ is the genetic variance for the treatment. All models were fitted using the flexible linear mixed modelling software ASReml-R [46] available in the R statistical computing environment [47].

### Vernalization (Vrn), photoperiod response (Ppd) and semi-dwarfing (Rht) gene marker assays

*Vrn* and *Rht8* PCR-marker amplicons were visualized by agarose gel electrophoresis. *Vrn* polymorphisms assayed are considered diagnostic of winter/spring alleles conditioning vernalization sensitivity/insensitivity. For *Vrn-A1*, primer pair BT468/BT486 located in the promoter-region [48] was used. For *Vrn-B1* and *Vrn-D1*, three-primer mixtures identifying insertion/deletion polymorphisms in intron-1 of these genes were used: (Intr1/B/F, Intr1/B/R3 and Intr1/B/R4), and (Intr1/D/F, Intr1/D//R3 and Intr1/D/R4), respectively [49]. The marker for *Rht8* was the microsatellite *gwm261* linked to *Rht8*; the 192 bp amplicon size has sometimes correlated with the *Rht8* dwarfing allele [50, 51]. PCRs contained 5 % dimethyl sulfoxide and used annealing/extension temperatures of 65 °C/68 °C for *Vrn-A1*, 50 °C/68 °C for *Vrn-B1*, 60 °C/68 °C for *Vrn-D1* and 55 °C/72 °C for *gwm261*.

*Ppd-B1*, *Ppd-D1*, *Rht-B1* and *Rht-D1* gene markers were assayed using competitive allele-specific PCR (KASP™) assays done using an automated SNPLine system and Kraken™ software (DNA LGC Limited, London, UK). Assays targeted a SNP in exon-3 of *Ppd-B1* distinguishing *Ppd-B1c* from other alleles [52], the 2,089 bp deletion upstream of the coding region of *Ppd-D1* characteristic of ‘Ciano 67’ type photoperiod insensitive *Ppd-D1* alleles [52] and the SNP mutations in the *Rht-B1* and *Rht-D1* genes resulting in gibberellic acid insensitive dwarfism [53]. Primers for *Ppd-D1*, *Rht-B1* and *Rht-D1* assays were CerealsDB [54] sets wMAS000024, wMAS000001 and wMAS000002, and those for *Ppd-B1* represented an unpublished assay kindly provided by David Laurie, John Innes Centre, UK.

### Genetic map construction and QTL analysis

The Drysdale × Waagan DH lines and parents were SNP-genotyped at the Department of Primary Industries, Centre for AgriBioscience, Vic, using the wheat Illumina 9,000 SNP array [34]. These data and scores for *Rht-B1*, *Rht-D1* and *Ppd-B1* markers were used to construct the Drysdale × Waagan molecular marker genetic map using R/qtl software [55] and the WGAIM package [56, 57].

Heterogeneity within each parent variety posed challenges to map construction. The heterogeneity was evidenced by the high proportion of differences between the two SNP-genotyped Drysdale selections (18 % of the 7,759 scorable markers; the two Waagan selections differed at 0.3 % of markers), the *Vrn-D1* marker heterogeneity observed across the 10 Waagan selections (6 and 4 lines carried the winter and spring allele, respectively; and this marker was monomorphic in the DH lines) and by markers that were monomorphic in all DH progeny of particular F_1_ plants. The latter markers tended to be clustered in particular chromosome segments (Additional file 8: Figure S1), suggesting that these segments were heterogeneous within the parent varieties. To prevent this from compromising the map, the 199 markers that were monomorphic in 4 to 8 of the 13 sub-populations had their marker scores converted to missing data in those sub-populations, and the 70 markers that were monomorphic in 9 to 12 of the sub-populations were eliminated from the analysis altogether.

Linkage groups were formed using a logarithm of odds (LOD) threshold of 5 and a maximum recombination frequency of 0.4. Associations of high LOD and high recombination frequency identified 26 markers assigned incorrect allele phase, and these were corrected. The “calc.errorlod” function with error LOD > 4 was applied to identify ‘singleton’ (likely error) scores that were subsequently removed. The “findDupMarkers” function in R/qtl, with the “exact.only = FALSE” setting, was used to find markers that had no differences in their available marker scores, identifying 551 sets of genetically non-redundant markers from the total 926 mapped markers. To utilize all available scores, consensus scores were determined for each set of co-localizing markers using the “fix.map” function of the WGAIM package and used to construct a map of 551 non-redundant loci.

Marker orders within linkage groups were refined using the “Ripple” function of R/qtl and the Kosambi mapping function [58] used to calculate cM distances. Maps of linkage groups aligned well to the wheat SNP consensus map of Cavanagh et al. [34] (not shown). The overall patterns of recombination fractions and linkage also indicated the marker order to be largely correct (Additional file 11: Figure S3).

BLUPs derived from the linear mixed model were used for QTL analysis. QTL analysis was performed separately for traits under either control or heat conditions, and for trait HSIs, for each experiment, using GenStat release 16 [59]. Initially, QTL analysis was performed using simple interval mapping, then the selected candidate QTL were used as co-factors for composite interval mapping (CIM), setting the minimum co-factor proximity to 30 cM. For CIM, a 10 cM maximum step size and an adjusted Bonferroni correction of a genome-wide significance level of α = 0.05 [60] was calculated, defining *p* < 0.000245 as the threshold for reporting QTL. QTL effects likely to be coming from the same locus were inferred based on proximity of the most highly-associated markers (<30 cM) and/or the same parent contributing the positive allele for the same or biologically-related trait within or across experiments. Map graphics were drawn using software R/qtl [55, 61] and MapChart 2.1 [62].

### Physical location of 3BS QTL from this and previous studies

Locations of QTL for heat tolerance related traits mapped to 3BS in this and previous studies were compared by determining locations of markers on the wheat 3B reference sequence, by using BLAST searches at the URGI website [63].

### Ethics

This study required no ethics approval.

### Availability of data and materials

Marker score data and the mapping population are available by negotiation. All other raw data are available upon request.

## Additional files

Additional file 1: Table S1. Measured temperatures (°C) in greenhouse in Experiments 1 and 2. (PDF 61 kb) Additional file 2: Table S2. Means ± SE for traits measured in the two experiments with the Drysdale × Waagan doubled haploid population and its parents. (PDF 74 kb) Additional file 3: Table S3. Heritabilities (H^2^) of the traits for each treatment/experiment in the Drysdale × Waagan DH lines. (PDF 70 kb) Additional file 4: Table S4. Genotypic correlations between trait potentials (values in control) and heat susceptibility indices (HSIs), in the two experiments, in the Drysdale × Waagan DH lines. (PDF 83 kb) Additional file 5: Table S5. Details of all QTL effects identified in the Drysdale × Waagan mapping population in the two heat tolerance experiments. (PDF 165 kb) Additional file 6: Table S6. Details of all QTL effects identified in the Drysdale × Waagan mapping population in the two heat tolerance experiments, with QTL effects arranged by trait/condition, then ordered by R^2^. (PDF 174 kb) Additional file 7: Table S7. Molecular marker genetic map of the Drysdale × Waagan doubled haploid population. (XLSX 87 kb) Additional file 8: Figure S1. Molecular marker genetic map made from the Drysdale × Waagan population of 139 F_1_-derived doubled haploid lines. The map is represented here using a set of 551 genetically non-redundant markers; an (R) after a marker indicates genetic redundancy with other markers. Linkage groups were ordered and oriented along each chromosome so they aligned to the wheat consensus SNP map of Cavanagh et al. [34] and so the end of the short arm was at the top. The numbers to the left of each linkage group show cM distances from the top of each linkage group. Markers in bold were monomorphic in 2 to 8 of the 13 DH sub-populations (derived from different F_1_ plants) and may therefore identify chromosome segments that were heterogeneous within a parent variety. Markers that showed segregation distortion are in colour, with blue and red indicating an excess of Drysdale or Waagan alleles, respectively. The significance levels of segregation distortion are represent by asterisks (**p* < 0.05, ***p* < 0.01 and ****p* < 0.001). (PDF 119 kb) Additional file 9: Table S8. Summary statistics, by chromosome, for the Drysdale × Waagan linkage map (PDF 73 kb) Additional file 10: Figure S2. QTL detected for heat susceptibility (HSI) and absolute trait values in the Drysdale × Waagan doubled haploid population. The numbers to the left of each molecular marker linkage group indicate cM distances from the top. Colored boxes represent peaks of QTL effects, defined in each case by the markers with –Log_10_(p) test statistic values within 1.5 of the peak value. Blue: QTL for HSIs; black: QTL for DTA and plant height; green, red, and brown: QTL detected for the absolute trait values under control, heat, and both control and heat conditions, respectively. Solid and hashed bars indicate QTL detected in both experiments or in one experiment only, except for single-experiment effects where the bar was too short to show hashing: QTL2-all-traits, QTL11-HChlR27, QTL11-ChlR27-heat, QTL11-HI-control and QTL29-FL-control. DTA, days from sowing to anthesis; DTM, days from sowing to maturity; GFD, grain-filling duration defined as days from anthesis to 95 % spike senescence; FLSe, days from anthesis to 95 % flag leaf senescence; GWS, grain weight spike^−1^; GNS, grain number spike^−1^; SGW, single grain weight; ShW, shoot dry weight; PH, plant height; ChlC10DAA, chlorophyll content 10 days after anthesis; ChlC13DAA, chlorophyll content 13 days after anthesis; AUSC, area under the SPAD curve; ChlR13, rate of chlorophyll change between 10 and 13 days after anthesis; ChlR27, rate of chlorophyll change based on the linear regression of the measurements, at 10, 13 and 27 days after anthesis; FL, flag leaf length; FW, flag leaf width; HI, harvest index. An ‘R’ next to a marker indicates that it represents a group of genetically redundant markers. (PDF 168 kb) Additional file 11: Figure S3. Heat-plot for final Drysdale × Waagan genetic map of 551 loci. Represented are recombination fractions (upper-left half of figure) and LOD scores for linkage (lower-right half of figure) for all pairs of genetically non-redundant markers. Markers are arranged in order and by chromosome or chromosome fragment, from chromosome 1A (left) to 7D (right). With progression through the colour series blue-green-yellow-red, LOD score increases and recombination fraction decreases. Alignment of the red signals along the diagonal indicates that the marker orders are largely correct. (PDF 404 kb)

## Abbreviations

AUSC: area under the SPAD curve
ChlC10DAA: chlorophyll content 10 days after anthesis
ChlC13DAA: chlorophyll content 13 days after anthesis
ChlC27DAA: chlorophyll content 27 days after anthesis
ChlR13: rate of chlorophyll change between 10 and 13 days after anthesis
ChlR27: rate of chlorophyll change based on the linear regression of the measurements, at 10, 13 and 27 days after anthesis
DAA: days after anthesis
DH: doubled haploid
DTA: days from sowing to anthesis
DTM: days from sowing to maturity defined as 95 % spike senescence
FL: flag leaf length
FLSe: days from anthesis to 95 % flag leaf senescence
FW: flag leaf width
GFD: grain-filling duration defined as days from anthesis to 95 % spike senescence
GNS: grain number spike^−1^
GWS: grain weight spike^−1^
HI: harvest index
HSI: Heat Susceptibility Index
PH: plant height
SGW: single grain weight
ShW: shoot dry weight
