# Supplementary material for: A QTL on the short arm of wheat (Triticum aestivum L.) chromosome 3B affects the stability of grain weight in plants exposed to a brief heat shock early in grain filling
Source: BMC Plant Biol. 2016 Apr 22;16:100. doi: 10.1186/s12870-016-0784-6 (PMC4841048; doi:10.1186/s12870-016-0784-6)
Supplement: Additional file 1: Table S1. — Measured temperatures (°C) in greenhouse in Experiments 1 and 2. (PDF 61 kb) [file 12870_2016_784_MOESM1_ESM.pdf]

Table S1. Measured temperatures (ºC) in greenhouse in Experiments 1 and 2.

		Ave. day temp.	Ave. night temp.	Ave. min. temp.	Ave. max. temp.	Min. temp.	Max. temp.	No. days over 30ºC
Expt. 1	March	24.1	19.9	18.5	28.8	17.7	33.5	2
	April	23.4	19.3	18.1	26.6	16.5	29.8	0
	May	22.3	18.8	17.7	25	16.3	26.8	0
	June	22.2	18.8	17.7	24.7	16.7	25.4	0
	July	22.3	18.7	17.7	25.1	17.5	29.5	0
	August	22.2	18.6	17.5	25	17.3	25.9	0
Expt. 2	July	22.3	18.6	17.6	26.1	17.5	29.7	0
	August	22.2	18.6	17.5	25	17.2	25.8	0
	September	22.8	18.8	17.6	26	17	29.9	0
	October	23.2	18.3	16.9	28.6	14.7	33.2	9
	November	24.7	19.2	17.7	29.4	14.9	33	11
	December	23.3	20.9	19.5	26.6	17.1	31	2

Anthesis and maturity occurred May-June and July-August in the first trial and in September-October and November-December in the second trial, respectively.
