# Supplementary material for: A QTL on the short arm of wheat (Triticum aestivum L.) chromosome 3B affects the stability of grain weight in plants exposed to a brief heat shock early in grain filling
Source: BMC Plant Biol. 2016 Apr 22;16:100. doi: 10.1186/s12870-016-0784-6 (PMC4841048; doi:10.1186/s12870-016-0784-6)
Supplement: Additional file 3: Table S3. — Heritabilities (H2) of the traits for each treatment/experiment in the Drysdale × Waagan DH lines. (PDF 70 kb) [file 12870_2016_784_MOESM3_ESM.pdf]

Table S3. Heritabilities (H^2) of the traits for each treatment/experiment in the Drysdale x Waagan DH lines

	Control		Heat	
**Trait**	**Expt. 1**	**Expt.2**	**Expt. 1**	**Expt.2**
**DTA**	0.61	0.66	0.59	0.68
**DTM**	0.6	0.67	0.55	0.63
**GFD**	0.65	0.72	0.71	0.79
**FLSe**	0.77	0.6	0.71	0.74
**GWS**	0.83	0.85	0.85	0.8
**GNS**	0.64	0.76	0.64	0.74
**SGW**	0.79	0.8	0.81	0.82
**ShW**	0.86	0.91	0.88	0.91
**PH**	0.96	0.96	0.96	0.96
**ChlC10DAA**	0.76	0.72	0.75	0.73
**ChlC13DAA**	0.65	0.61	0.84	0.84
**ChlC27DAA**	0.65	0.39	0.87	0.82
**AUSC**	0.71	0.5	0.87	0.82
**ChlR13**	0.00	0.00	0.87	0.87
**ChlR27**	0.25	0.33	0.84	0.81
**FL**	0.49	0.5	0.45	0.5
**FW**	0.6	0.71	0.6	0.68
**HI**	0.3	0.78	0.44	0.76

DTA, days from sowing to anthesis; DTM, days from sowing to maturity defined as 95% spike senescence; GFD, grain-filling duration defined as days from anthesis to 95% spike senescence; FLSe, days from anthesis to 95% flag leaf senescence; GWS, grain weight spike^{-1}; GNS, grain number spike^{-1}; SGW, single grain weight; ShW, shoot dry weight; PH, plant height; ChlC10DAA, chlorophyll content 10 days after anthesis, i.e., just before heat treatment period; ChlC13DAA, chlorophyll content 13 days after anthesis, i.e., just after heat treatment period; AUSC, area under the SPAD curve made from measurements at 10, 13 and 27 days after anthesis, i.e., incorporates the period during heat treatment and two weeks after; ChlR13, rate of chlorophyll change between 10 and 13 days after anthesis, i.e., during the heat treatment period; ChlR27, rate of chlorophyll change based on the linear regression of the measurements, at 10, 13 and 27 days after anthesis; FL, flag leaf length; FW, flag leaf width; HI, harvest index.

Traits are partitioned in the table based on their relationships to duration of development phases, yield components and biomass, chlorophyll content and stability, flag leaf dimensions and harvest index, respectively.

Values are shaded according to magnitude to aid visualization.
