# Supplementary material for: A QTL on the short arm of wheat (Triticum aestivum L.) chromosome 3B affects the stability of grain weight in plants exposed to a brief heat shock early in grain filling
Source: BMC Plant Biol. 2016 Apr 22;16:100. doi: 10.1186/s12870-016-0784-6 (PMC4841048; doi:10.1186/s12870-016-0784-6)
Supplement: Additional file 4: Table S4. — Genotypic correlations between trait potentials (values in control) and heat susceptibility indices (HSIs), in the two experiments, in the Drysdale × Waagan DH lines. (PDF 83 kb) [file 12870_2016_784_MOESM4_ESM.pdf]

Table S4. Genotypic correlations between trait potentials (values in control) and heat susceptibility indices (HSIs), in the two experiments, in the Drysdale x Waagan DH lines.

			Trait potentials															
		Trait	DTA	DTM	GFD	FLSe	GWS	GNS	SGW	ShW	PH	ChlC10 DAA	ChlC13 DAA	ChlC27 DAA	AUSC	ChlR13	ChlR27	FW	FL
HSIs	Expt. 1	DTM	0.17*	0.53***	0.33***	0.26**	-0.17*	-0.14	-0.12	-0.20*	-0.18*	-0.17*	-0.20*	-0.13	-0.17*	-0.1	0.05	0.23**	0.04
		GFD	0.19*	0.34***	0.31***	0.35***	-0.26**	-0.20*	-0.20*	-0.34***	-0.31***	-0.13	-0.18*	-0.12	-0.16	-0.25**	-0.07	0.28***	0.12
		FLSe	0.27**	0.35***	0.30***	0.60***	-0.25**	-0.13	-0.27**	-0.32***	-0.31***	-0.16*	-0.22**	-0.15	-0.20*	-0.25**	0.01	0.33***	0.22**
		GWS	0.09	0.14	-0.01	0.02	0	-0.01	0.02	-0.07	-0.05	-0.06	-0.1	-0.06	-0.07	-0.18*	-0.06	0.08	0.06
		SGW	0.13	0.23**	0.11	0.06	-0.14	-0.29***	0.13	-0.18*	-0.08	-0.14	-0.17*	-0.14	-0.15	-0.15	-0.08	0.02	0.02
		ShW	0.1	0.06	-0.04	0.03	-0.07	0	-0.11	-0.05	-0.11	-0.05	-0.09	-0.06	-0.07	-0.15	0.01	0.06	-0.03
		ChlC13DAA	-0.11	0.02	0.05	0.03	-0.13	-0.14	0.01	-0.11	0.01	-0.32***	-0.35***	-0.33***	-0.35***	-0.08	0.02	-0.05	0.04
		ChlC27DAA	-0.07	0.06	0.13	0.12	-0.24**	-0.23**	-0.08	-0.26**	-0.11	-0.24**	-0.28***	-0.15	-0.22**	-0.07	0.04	-0.07	0.02
		AUSC	-0.13	0.03	0.09	0.06	-0.18	-0.17*	-0.05	-0.19*	-0.05	-0.25**	-0.30***	-0.21	-0.25**	-0.06	0.05	-0.05	0
		ChlR13	-0.04	0.1	0.05	0.02	-0.08	-0.15	0.08	-0.07	0.08	-0.37***	-0.42***	-0.39**	-0.42***	-0.17*	0	-0.11	0.09
		ChlR27	-0.07	-0.02	-0.03	-0.07	-0.01	-0.05	0.06	-0.01	0.15	0.07	0.05	0.03	0.05	-0.1	-0.1	-0.19*	-0.11
	Expt. 2	DTM	0.17*	0.49***	0.05	-0.05	0.17*	0.18*	0.05	0.16	0.13	-0.12	-0.12	-0.19*	-0.18*	-0.07	-0.24**	-0.02	-0.04
		GFD	0.15	0.29***	-0.04	-0.22**	0.33***	0.33***	0.14	0.34***	0.30***	0.03	-0.02	-0.24**	-0.15	-0.21*	-0.53***	-0.01	-0.05
		FLSe	0.05	0.1	-0.08	0.02	0.19*	0.18*	0.06	0.15	0.1	0.16	0.12	0.02	0.08	-0.14	-0.49***	0.14	0.05
		GWS	0.31***	0.26**	-0.04	-0.25**	0.46***	0.49***	0.15	0.23**	0.13	-0.13	-0.18*	-0.38***	-0.30***	-0.21*	-0.47***	0.12	-0.04
		SGW	0.23**	0.20*	0.03	-0.16	0.14	0.1	0.19*	0.07	0.03	-0.26**	-0.31***	-0.41***	-0.40***	-0.26**	-0.57***	0.03	-0.01
		ShW	0.13	0.08	-0.02	-0.13	0.1	0.11	0.07	0.11	0.05	-0.11	-0.12	-0.21*	-0.18*	-0.06	-0.15	-0.02	-0.15
		ChlC13DAA	-0.1	-0.06	0.11	0.05	0.01	-0.03	0.13	-0.01	0.13	-0.32***	-0.32***	-0.30***	-0.34***	-0.11	-0.49***	-0.19*	0.13
		ChlC27DAA	0.18*	0.16	-0.08	-0.19*	0.27***	0.23**	0.23**	0.20*	0.22**	-0.25**	-0.31***	-0.37***	-0.38***	-0.30***	-0.86***	-0.07	0.12
		AUSC	0.06	0.07	0	-0.08	0.17*	0.13	0.19*	0.11	0.18	-0.29***	-0.33***	-0.30***	-0.34***	-0.21*	-0.71***	-0.11	0.14
		ChlR13	0.05	0.08	0.03	-0.07	0.12	0.1	0.14	0.05	0.15	-0.36***	-0.38***	-0.42***	-0.44***	-0.24**	-0.60***	-0.09	0.11
		ChlR27	0.42***	0.33***	-0.24**	-0.41***	0.49***	0.48***	0.29***	0.36***	0.31***	-0.08	-0.19*	-0.54***	-0.4***	-0.43***	-0.84***	0.03	0.07

Values are Pearson correlation coefficients, and significance level indicated by *p < 0.05, **p < 0.01 and ***p < 0.001.

Significant positive and negative correlations are highlighted by the green and peach shading, respectively, to aid visualization.

Positive values mean the lower trait value under control conditions was correlated with tolerance.

Greyed out cells represent comparisons between arithmetically related variables where the direction of the correlation (significant or not) is consistent with the possibility that the correlation was a mathematical artefact.

DTA, days from sowing to anthesis; DTM, days from sowing to maturity defined as 95% spike senescence; GFD, grain-filling duration defined as days from anthesis to 95% spike senescence; FLSe, days from anthesis to 95% flag leaf senescence; GWS, grain weight spike^{-1} (g); GNS, grain number spike^{-1}; SGW, single grain weight (mg); ShW, shoot dry weight (g); PH, plant height (cm); ChlC10DAA, chlorophyll content 10 days after anthesis, i.e., just before heat treatment period (SPAD units); ChlC13DAA, chlorophyll content 13 days after anthesis, i.e., just after heat treatment period (SPAD units); AUSC, area under the SPAD curve made from measurements at 10, 13 and 27 days after anthesis, i.e., incorporates the period during-heat treatment and 2-weeks after; ChlR13, rate of chlorophyll change between 10 and 13 days after anthesis, i.e., during the heat treatment period (SPAD units day^{-1}); ChlR27, rate of chlorophyll change based on the linear regression of the measurements, at 10, 13 and 27 days after anthesis (SPAD units day^{-1}); FL, flag leaf length (cm); FW, flag leaf width (cm).

HSIs for DTA, GNS, PH, Chl10DAA FW and FL were not used in the correlation analysis as the heat treatments had no significant effect on these traits.

Traits are partitioned in the table based on their relationships to duration of development phases, yield components and biomass, chlorophyll content and stability, flag leaf dimensions and harvest index, respectively.
