# Supplementary material for: A QTL on the short arm of wheat (Triticum aestivum L.) chromosome 3B affects the stability of grain weight in plants exposed to a brief heat shock early in grain filling
Source: BMC Plant Biol. 2016 Apr 22;16:100. doi: 10.1186/s12870-016-0784-6 (PMC4841048; doi:10.1186/s12870-016-0784-6)
Supplement: Additional file 5: Table S5. — Details of all QTL effects identified in the Drysdale × Waagan mapping population in the two heat tolerance experiments. (PDF 165 kb) [file 12870_2016_784_MOESM5_ESM.pdf]

Table S5. Details of all QTL effects identified in the Drysdale x Waagan mapping population in the two heat tolerance experiments.

QTL	Trait	Condition	Expt.	Linkage group	cM position	Most closely-associated marker(s)	Positive allele	Test statistic $-Log_{10}(p)$	R^2	Additive effect
QTL1	GFD	Control	2	1A1	59.7	wsnp_Ex_c1997_3756118	D	6.3	7.3	0.52
QTL1	FLSe	Control	2	1A1	56.1	wsnp_Ku_c10292_17066821	D	3.8	5.4	0.89
QTL1	SGW	Control	1	1A1	36.5	wsnp_Ex_c200_391493	W	4.3	5.6	1.07
QTL1	PH	Control	2	1A1	66.2	wsnp_BE517729A_Ta_2_1	W	4.2	1.1	1.94
QTL1	ShW	Heat	2	1A1	48.2	wsnp_Ku_c23926_33870364	W	4.9	2.0	0.08
QTL1	PH	Heat	2	1A1	67.6	wsnp_Ex_c5060_8985678	W	4.9	1.5	2.31
QTL2	GWS	Control	2	1A2	0.0	wsnp_Ku_c40759_48907151	D	3.9	6.7	0.17
QTL2	GNS	Control	2	1A2	0.0	wsnp_Ku_c40759_48907151	D	4.7	7.9	2.73
QTL2	ChlR27	Control	2	1A2	0.0	wsnp_Ku_c40759_48907151	W	4.6	11.3	0.04
QTL2	ChlC10DAA	Control (pre-heat)	1	1A2	0.0	wsnp_Ku_c40759_48907151	D	3.8	4.1	0.34
QTL2	ChlR27	Heat	2	1A2	0.0	wsnp_Ku_c40759_48907151	W	4.4	10.7	0.10
QTL2	ChlR27	HSI	2	1A2	0.0	wsnp_Ku_c40759_48907151	D	5.9	11.0	0.30
QTL3	SGW	Heat	2	1B	83.4	wsnp_Ku_c18227_27490539	W	4.3	6.8	0.97
QTL4	FL	Control (pre-heat)	1	2A	0.0	wsnp_Ex_c2772_5130007	W	3.9	7.6	0.30
QTL5	ChlC13DAA	Control	2	2A	78.0	wsnp_Ex_rep_c102538_87682273	W	5.1	10.0	0.49
QTL5	ChlC27DAA	Control	1	2A	75.0	wsnp_Ex_c42720_49228237	W	5.0	9.0	0.48
QTL5	ChlC27DAA	Control	2	2A	80.9	wsnp_Ra_c4503_8155485	W	5.2	9.2	0.43
QTL5	AUSC	Control	1	2A	75.0	wsnp_Ex_c42720_49228237	W	4.5	8.1	7.88
QTL5	AUSC	Control	2	2A	80.9	wsnp_Ra_c4503_8155485	W	5.9	10.5	7.86
QTL5	ChlC10DAA	Control (pre-heat)	1	2A	75.0	wsnp_Ex_c42720_49228237	W	4.0	4.1	0.34
QTL5	ChlC10DAA	Control (pre-heat)	2	2A	108.4	wsnp_Ex_c3808_6924802	W	4.3	8.7	0.47
QTL5	FW	Control (pre-heat)	2	2A	109.9	wsnp_Ex_c59095_60108185	W	5.4	6.8	0.03
QTL5	DTM	Heat	1	2A	88.4	wsnp_Ex_c5984_10493714	W	3.8	7.1	0.69
QTL5	PH	Heat	1	2A	60.1	wsnp_BQ168780B_Ta_2_1	D	4.3	1.3	2.08
QTL6	ChlC27DAA	Control	2	2B1	0.0	wsnp_Ra_c14112_22155451	W	6.0	11.5	0.48
QTL6	AUSC	Control	2	2B1	0.0	wsnp_Ra_c14112_22155451	W	4.3	7.9	6.83
QTL6	DTA	Control (pre-heat)	2	2B1	5.4	wsnp_Ra_c14112_22155451	D	3.8	6.9	0.72
QTL7	FL	Control (pre-heat)	1	2B1	133.1	wsnp_RFL_Contig1892_1042675	D	6.4	13.9	0.41
QTL7	FL	Control (pre-heat)	2	2B1	126.3	wsnp_JD_c6010_7167159	D	6.2	13.5	0.43
QTL8	ChlC13DAA	Control	1	2D3	0.0	wsnp_Ex_c2258_4232538	D	8.3	12.7	0.55
QTL8	ChlC27DAA	Control	1	2D3	0.0	wsnp_Ex_c2258_4232538	D	6.4	11.6	0.55
QTL8	AUSC	Control	1	2D3	0.0	wsnp_Ex_c2258_4232538	D	7.1	13.3	10.09
QTL8	ChlC10DAA	Control (pre-heat)	1	2D3	0.0	wsnp_Ex_c2258_4232538	D	8.3	14.0	0.63
QTL8	ShW	Heat	2	2D3	3.6	wsnp_JD_c5919_7081809	D	4.6	3.2	0.10
QTL8	ChlC13DAA	Heat	1	2D3	5.8	wsnp_Ex_c7260_12463738	D	6.1	8.5	0.91
QTL8	ChlC27DAA	Heat	1	2D3	5.0	wsnp_Ex_c20011_29041563	D	5.4	6.7	0.87
QTL8	AUSC	Heat	1	2D3	5.8	wsnp_Ex_c7260_12463738	D	6.4	8.1	15.04
QTL9	GWS	Control	1	2D4	25.6	wsnp_Ku_c30494_40319867	D	5.4	7.9	0.09
QTL9	SGW	Control	2	2D4	32.4	wsnp_RFL_Contig2659_2346243	D	3.5	6.1	0.86
QTL9	ShW	Control	1	2D4	25.6	wsnp_Ku_c30494_40319867	D	4.0	5.6	0.08
QTL9	ShW	Control	2	2D4	18.8	wsnp_Ku_c30494_40319867	D	4.0	2.8	0.09
QTL9	GWS	Heat	1	2D4	25.6	wsnp_Ku_c30494_40319867	D	5.5	7.8	0.09
QTL9	SGW	Heat	2	2D4	32.4	wsnp_RFL_Contig2659_2346243	D	3.5	7.5	1.02
QTL9	ShW	Heat	1	2D4	25.6	wsnp_Ku_c30494_40319867	D	5.4	4.3	0.07
QTL10	PH	Control	2	3A2	15.3	wsnp_Ex_c4069_7354375	D	7.1	1.5	2.27
QTL10	FW	Control (pre-heat)	2	3A2	30.4	wsnp_Ex_c25668_34932304	D	5.4	6.0	0.02
QTL10	ShW	Heat	1	3A2	15.3	wsnp_Ex_c4069_7354375	D	3.9	2.0	0.05
QTL10	PH	Heat	1	3A2	24.0	wsnp_Ex_c1141_2191485	D	3.9	1.1	1.94
QTL10	PH	Heat	2	3A2	15.3	wsnp_Ex_c4069_7354376	D	6.6	2.1	2.75

Table S5 (continued)

QTL	Trait	Condition	Expt.	Linkage group	cM position	Most closely-associated marker(s)	Positive allele	Test statistic $-Log_{10}(p)$	R^2	Additive effect
QTL11	ChlC13DAA	Control	1	3B1	1.4	wsnp_Ex_c12875_20407926	W	7.7	14.6	0.59
QTL11	ChlC13DAA	Control	2	3B1	1.4	wsnp_Ex_c12875_20407926	W	8.9	19.4	0.67
QTL11	ChlC27DAA	Control	1	3B1	0.0	wsnp_Ra_c41135_48426638	W	8.6	17.1	0.66
QTL11	ChlC27DAA	Control	2	3B1	0.0	wsnp_Ra_c41135_48426638	W	11.5	23.0	0.68
QTL11	AUSC	Control	1	3B1	0.0	wsnp_Ra_c41135_48426638	W	7.5	15.2	10.78
QTL11	AUSC	Control	2	3B1	1.4	wsnp_Ex_c12875_20407926	W	10.5	20.4	10.98
QTL11	HI	Control	2	3B1	0.0	wsnp_Ra_c41135_48426638	D	8.7	11.5	0.94
QTL11	ChlC10DAA	Control (pre-heat)	1	3B1	1.4	wsnp_Ex_c12875_20407926	W	10.7	17.9	0.71
QTL11	ChlC10DAA	Control (pre-heat)	2	3B1	1.4	wsnp_Ex_c12875_20407926	W	7.4	16.8	0.65
QTL11	GFD	Heat	1	3B1	1.4	wsnp_Ex_c12875_20407926	W	6.4	13.1	0.76
QTL11	FLSe	Heat	1	3B1	3.2	wsnp_BE497169B_Ta_2_1	W	5.5	14.2	2.06
QTL11	GWS	Heat	1	3B1	11.0	wsnp_BE497169B_Ta_2_1	W	7.1	11.2	0.11
QTL11	SGW	Heat	1	3B1	11.0	wsnp_BE497169B_Ta_2_1	W	7.0	11.5	1.65
QTL11	ShW	Heat	1	3B1	11.0	wsnp_BE497169B_Ta_2_1	W	17.9	14.2	0.14
QTL11	ShW	Heat	2	3B1	40.5	wsnp_Ku_c3817_7009093/ wsnp_Ex_c44375_50444756	W	5.8	3.5	0.10
QTL11	ChlC13DAA	Heat	1	3B1	1.4	wsnp_Ex_c12875_20407926	W	26.0	42.0	2.01
QTL11	ChlC13DAA	Heat	2	3B1	0.0	wsnp_Ra_c41135_48426638	W	16.0	34.3	1.95
QTL11	ChlC27DAA	Heat	1	3B1	1.4	wsnp_Ex_c12875_20407926	W	36.3	54.4	2.49
QTL11	ChlC27DAA	Heat	2	3B1	0.0	wsnp_Ra_c41135_48426638	W	8.0	19.9	2.55
QTL11	AUSC	Heat	1	3B1	1.4	wsnp_Ex_c12875_20407926	W	33.2	48.6	36.94
QTL11	AUSC	Heat	2	3B1	0.0	wsnp_Ra_c41135_48426638	W	13.3	28.0	34.31
QTL11	ChlR13	Heat	1	3B1	0.0	wsnp_Ra_c41135_48426638	W	19.7	39.6	0.37
QTL11	ChlR13	Heat	2	3B1	0.0	wsnp_Ra_c41135_48426638	W	13.2	27.2	0.37
QTL11	ChlR27	Heat	1	3B1	0.0	wsnp_Ra_c41135_48426638	W	29.5	50.4	0.07
QTL11	GFD	HSI	1	3B1	3.2	wsnp_BE497169B_Ta_2_1	D	4.4	10.0	0.12
QTL11	FLSe	HSI	1	3B1	1.4	wsnp_Ex_c12875_20407926	D	6.7	14.4	0.27
QTL11	GWS	HSI	1	3B1	3.2	wsnp_BE497169B_Ta_2_1	D	8.8	21.6	1.16
QTL11	GWS	HSI	2	3B1	0.0	wsnp_Ra_c41135_48426638	D	5.9	15.0	0.38
QTL11	SGW	HSI	1	3B1	3.2	wsnp_BE497169B_Ta_2_1	D	8.1	20.1	0.92
QTL11	SGW	HSI	2	3B1	1.4	wsnp_Ex_c12875_20407926	D	4.7	10.8	0.16
QTL11	ShW	HSI	1	3B1	3.2	wsnp_BE497169B_Ta_2_1	D	9.3	22.8	1.62
QTL11	ChlC13DAA	HSI	1	3B1	1.4	wsnp_Ex_c12875_20407926	D	16.6	35.7	0.67
QTL11	ChlC13DAA	HSI	2	3B1	0.0	wsnp_Ra_c41135_48426638	D	10.0	23.9	0.81
QTL11	ChlC27DAA	HSI	1	3B1	1.4	wsnp_Ex_c12875_20407926	D	21.3	38.6	1.28
QTL11	ChlC27DAA	HSI	2	3B1	0.0	wsnp_Ra_c41135_48426638	D	5.3	13.4	0.62
QTL11	AUSC	HSI	1	3B1	1.4	wsnp_Ex_c12875_20407926	D	20.5	38.3	0.91
QTL11	AUSC	HSI	2	3B1	0.0	wsnp_Ra_c41135_48426638	D	7.3	18.3	0.67
QTL11	ChlR13	HSI	1	3B1	0.0	wsnp_Ra_c41135_48426638	D	16.3	39.7	0.55
QTL11	ChlR13	HSI	2	3B1	0.0	wsnp_Ra_c41135_48426638	D	13.2	27.2	0.80
QTL11	ChlR27	HSI	2	3B1	0.0	wsnp_Ra_c41135_48426638	D	9.3	19.4	0.40
QTL11	HI	HSI	2	3B1	0.0	wsnp_Ra_c41135_48426638	D	4.1	10.3	0.29
QTL12	SGW	Heat	2	3B2	54.9	wsnp_Ex_c1097_2105209	W	3.8	5.9	0.91
QTL13	GFD	Control	1	4A1	0.0	wsnp_Ex_c11474_18507872	D	4.7	9.4	0.53
QTL13	ChlC27DAA	HSI	1	4A1	0.0	wsnp_Ex_c11474_18507872	D	4.7	7.4	0.56
QTL13	AUSC	HSI	1	4A1	0.0	wsnp_Ex_c11474_18507872	D	3.8	6.1	0.36
QTL14	SGW	Heat	1	4A2	0.0	wsnp_Ex_c41074_47987860	W	4.7	5.1	1.11
QTL14	ShW	Heat	1	4A2	0.0	wsnp_Ex_c41074_47987860	W	3.8	1.4	0.04
QTL15	PH	Control	1	4A2	41.6	wsnp_Ex_rep_c68569_67411985	W	5.6	2.2	2.74
QTL15	PH	Control	2	4A2	42.3	wsnp_RFL_Contig25_2082245	W	9.0	1.8	2.52
QTL15	FW	Control (pre-heat)	2	4A2	47.3	wsnp_Ex_c55245_57821389	D	4.7	6.0	0.02
QTL15	GFD	Heat	2	4A2	42.3	wsnp_RFL_Contig25_2082245	D	4.6	4.2	0.49
QTL15	PH	Heat	1	4A2	41.6	wsnp_Ex_rep_c68569_67411985	W	6.0	1.8	2.47
QTL15	PH	Heat	2	4A2	42.3	wsnp_RFL_Contig25_2082245	W	7.3	2.3	2.90
QTL15	GFD	HSI	2	4A2	42.3	wsnp_RFL_Contig25_2082245	W	3.8	8.4	0.12

Table S5 (continued)

QTL	Trait	Condition	Expt.	Linkage group	cM position	Most closely-associated marker(s)	Positive allele	Test statistic $-Log_{10}(p)$	R^2	Additive effect
QTL16	DTM	Control	1	4B	29.6	wsnp_Ex_c17561_26284693	W	5.0	10.7	0.96
QTL17 (Rht-B1)	GFD	Control	2	4B	86.3	wsnp_RFL_Contig4151_4728831	W	14.9	17.1	0.79
QTL17 (Rht-B1)	FLSe	Control	1	4B	83.9	Rht-B1	W	6.6	12.2	2.40
QTL17 (Rht-B1)	FLSe	Control	2	4B	86.3	wsnp_RFL_Contig4151_4728831	W	7.4	11.9	1.32
QTL17 (Rht-B1)	GWS	Control	1	4B	83.9	Rht-B1	D	12.0	15.2	0.12
QTL17 (Rht-B1)	SGW	Control	1	4B	83.9	Rht-B1	D	14.2	21.5	2.09
QTL17 (Rht-B1)	SGW	Control	2	4B	83.9	Rht-B1	D	10.5	15.8	1.38
QTL17 (Rht-B1)	ShW	Control	1	4B	83.9	Rht-B1	D	16.8	18.6	0.14
QTL17 (Rht-B1)	ShW	Control	2	4B	83.9	Rht-B1	D	19.6	20.7	0.24
QTL17 (Rht-B1)	PH	Control	1	4B	83.9	Rht-B1	D	37.6	32.9	10.58
QTL17 (Rht-B1)	PH	Control	2	4B	83.9	Rht-B1	D	83.9	37.1	11.44
QTL17 (Rht-B1)	HI	Control	2	4B	77.7	wsnp_Ex_c18433_27269748/ Rht-B1	W	15.8	23.8	1.35
QTL17 (Rht-B1)	DTM	Heat	2	4B	83.9	Rht-B1	W	3.8	5.9	0.62
QTL17 (Rht-B1)	GFD	Heat	2	4B	83.9	Rht-B1	W	11.1	21.2	1.11
QTL17 (Rht-B1)	GWS	Heat	1	4B	83.9	Rht-B1	D	12.5	15.1	0.13
QTL17 (Rht-B1)	GWS	Heat	2	4B	83.9	Rht-B1	D	3.8	5.7	0.12
QTL17 (Rht-B1)	SGW	Heat	1	4B	86.3	wsnp_RFL_Contig4151_4728831	D	26.0	33.0	2.81
QTL17 (Rht-B1)	SGW	Heat	2	4B	83.9	Rht-B1	D	6.1	10.2	1.19
QTL17 (Rht-B1)	ShW	Heat	1	4B	83.9	Rht-B1	D	34.2	23.1	0.17
QTL17 (Rht-B1)	ShW	Heat	2	4B	83.9	Rht-B1	D	32.6	26.5	0.28
QTL17 (Rht-B1)	PH	Heat	1	4B	83.9	Rht-B1	D	46.3	36.8	11.19
QTL17 (Rht-B1)	PH	Heat	2	4B	83.9	Rht-B1	D	44.9	35.9	11.38
QTL17 (Rht-B1)	HI	Heat	2	4B	77.7	wsnp_Ex_c18433_27269748/ Rht-B1	W	12.0	25.4	1.22
QTL18	DTM	Control	1	4B	135.5	wsnp_Ex_c4148_7495656	W	4.6	7.9	0.83
QTL18	DTM	Control	2	4B	135.5	wsnp_Ex_c4148_7495656	W	8.8	19.2	1.25
QTL18	GFD	Control	2	4B	135.5	wsnp_Ex_c4148_7495656	D	9.4	8.7	0.57
QTL18	FLSe	Control	2	4B	135.5	wsnp_Ex_c4148_7495656	D	16.4	27.9	2.02
QTL18	GWS	Control	1	4B	135.5	wsnp_Ex_c4148_7495656	W	7.8	9.5	0.09
QTL18	GWS	Control	2	4B	135.5	wsnp_Ex_c4148_7495656	W	7.1	12.5	0.23
QTL18	GNS	Control	1	4B	135.5	wsnp_Ex_c4148_7495656	W	5.5	10.3	1.48
QTL18	GNS	Control	2	4B	135.5	wsnp_Ex_c4148_7495656	W	9.0	15.5	3.83
QTL18	ShW	Control	1	4B	135.5	wsnp_Ex_c4148_7495656	W	4.4	5.1	0.07
QTL18	ShW	Control	2	4B	135.5	wsnp_Ex_c4148_7495658	W	11.1	9.8	0.17
QTL18	DTA	Control (pre-heat)	1	4B	135.5	wsnp_Ex_c4148_7495656	W	21.7	37.1	1.48
QTL18	DTA	Control (pre-heat)	2	4B	135.5	wsnp_Ex_c4148_7495656	W	20.5	35.9	1.65
QTL18	FL	Control (pre-heat)	2	4B	126.1	wsnp_Ex_c39876_47057394	D	6.9	13.6	0.44
QTL18	FW	Control (pre-heat)	1	4B	108.3	wsnp_CAP12_rep_c4278_1949864	W	10.1	20.4	0.03
QTL18	FW	Control (pre-heat)	2	4B	117.2	wsnp_Ex_c39876_47057394	W	16.9	31.2	0.06
QTL18	DTM	Heat	1	4B	141.3	wsnp_BE403378B_Ta_2_1	W	7.1	14.0	0.97
QTL18	DTM	Heat	2	4B	135.5	wsnp_Ex_c4148_7495656	W	12.8	22.2	1.21
QTL18	GFD	Heat	1	4B	108.3	wsnp_CAP12_rep_c4278_1949864	D	7.5	19.8	0.93
QTL18	GFD	Heat	2	4B	127.5	wsnp_Ku_c11570_18860306	D	5.4	12.9	0.87
QTL18	FLSe	Heat	2	4B	149.9	wsnp_BE403378B_Ta_2_1/ wsnp_CAP7_c5487_2464794	D	5.7	14.8	2.15
QTL18	GWS	Heat	1	4B	135.5	wsnp_Ex_c4148_7495656	W	4.1	4.4	0.07
QTL18	GWS	Heat	2	4B	149.9	wsnp_BE403378B_Ta_2_1	W	7.1	15.6	0.20
QTL18	GNS	Heat	1	4B	135.5	wsnp_Ex_c4148_7495656	W	3.9	4.7	0.98
QTL18	GNS	Heat	2	4B	135.5	wsnp_Ex_c4148_7495656	W	9.6	17.5	3.71
QTL18	ShW	Heat	1	4B	135.5	wsnp_Ex_c4148_7495657	W	5.0	2.6	0.06
QTL18	ShW	Heat	2	4B	135.5	wsnp_Ex_c4148_7495659	W	12.7	10.3	0.17
QTL18	GFD	HSI	1	4B	99.5	wsnp_CAP12_rep_c4278_1949864	W	5.5	12.4	0.13
QTL18	FLSe	HSI	1	4B	117.2	wsnp_Ex_c39876_47057394	W	6.5	17.7	0.30
QTL18	ChlR27	HSI	2	4B	135.5	wsnp_Ex_c4148_7495656	W	4.7	8.4	0.26

Table S5 (continued)

QTL	Trait	Condition	Expt.	Linkage group	cM position	Most closely-associated marker(s)	Positive allele	Test statistic $-Log_{10}(p)$	R^2	Additive effect
QTL19 (Rht-D1)	GFD	Control	2	4D	0.0	Rht-D1	D	10.8	12.1	0.66
QTL19 (Rht-D1)	FLSe	Control	1	4D	0.0	Rht-D1	D	6.2	11.3	2.30
QTL19 (Rht-D1)	FLSe	Control	2	4D	0.0	Rht-D1	D	12.5	19.1	1.68
QTL19 (Rht-D1)	GWS	Control	1	4D	0.0	Rht-D1	W	29.6	37.0	0.19
QTL19 (Rht-D1)	GWS	Control	2	4D	0.0	Rht-D1	W	8.4	15.4	0.26
QTL19 (Rht-D1)	GNS	Control	1	4D	0.0	Rht-D1	W	10.7	21.4	2.13
QTL19 (Rht-D1)	GNS	Control	2	4D	0.0	Rht-D1	W	7.5	13.1	3.53
QTL19 (Rht-D1)	SGW	Control	1	4D	0.0	Rht-D1	W	8.0	11.5	1.27
QTL19 (Rht-D1)	SGW	Control	2	4D	0.0	Rht-D1	W	9.1	12.9	1.25
QTL19 (Rht-D1)	ShW	Control	1	4D	0.0	Rht-D1	W	39.9	47.3	0.23
QTL19 (Rht-D1)	ShW	Control	2	4D	0.0	Rht-D1	W	50.5	48.7	0.37
QTL19 (Rht-D1)	PH	Control	1	4D	0.0	Rht-D1	W	50.0	56.5	13.87
QTL19 (Rht-D1)	PH	Control	2	4D	0.0	Rht-D1	W	62.3	56.4	14.11
QTL19 (Rht-D1)	ChlC27DAA	Control	2	4D	2.9	wsnp_CAP11_c356_280910	D	3.7	6.3	0.36
QTL19 (Rht-D1)	HI	Control	2	4D	0.0	Rht-D1	D	8.4	10.6	0.90
QTL19 (Rht-D1)	DTM	Heat	2	4D	0.0	Rht-D1	D	5.4	8.2	0.73
QTL19 (Rht-D1)	GFD	Heat	2	4D	0.0	Rht-D1	D	10.1	14.7	0.92
QTL19 (Rht-D1)	FLSe	Heat	2	4D	0.0	Rht-D1	D	5.5	11.6	1.90
QTL19 (Rht-D1)	GWS	Heat	1	4D	0.0	Rht-D1	W	23.8	28.7	0.18
QTL19 (Rht-D1)	GWS	Heat	2	4D	0.0	Rht-D1	W	12.4	23.1	0.25
QTL19 (Rht-D1)	GNS	Heat	1	4D	8.0	wsnp_Ex_rep_c107564_91144523	W	8.2	16.5	1.85
QTL19 (Rht-D1)	GNS	Heat	2	4D	0.0	Rht-D1	W	10.3	18.9	3.86
QTL19 (Rht-D1)	SGW	Heat	1	4D	0.0	Rht-D1	W	7.6	10.0	1.17
QTL19 (Rht-D1)	SGW	Heat	2	4D	0.0	Rht-D1	W	5.8	9.5	1.15
QTL19 (Rht-D1)	ShW	Heat	1	4D	0.0	Rht-D1	W	58.9	37.9	0.22
QTL19 (Rht-D1)	ShW	Heat	2	4D	0.0	Rht-D1	W	55.0	42.5	0.35
QTL19 (Rht-D1)	PH	Heat	1	4D	0.0	Rht-D1	W	56.5	56.4	13.86
QTL19 (Rht-D1)	PH	Heat	2	4D	0.0	Rht-D1	W	54.0	52.6	13.78
QTL19 (Rht-D1)	HI	Heat	2	4D	0.0	Rht-D1	D	10.8	19.4	1.07
QTL20	ChlC13DAA	Control	1	5A2	11.9	wsnp_Ex_c1481_2831499	D	6.7	8.6	0.45
QTL20	ChlC10DAA	Control (pre-heat)	1	5A2	3.8	wsnp_Ex_c1481_2831499	D	6.3	12.1	0.59
QTL20	ChlC13DAA	Heat	1	5A2	1.5	wsnp_JD_c43389_30288993	D	4.6	6.3	0.78
QTL20	AUSC	Heat	1	5A2	1.5	wsnp_JD_c43389_30288993	D	4.4	5.4	12.28
QTL20	HI	Heat	1	5A2	0.0	wsnp_CAP11_c923_558715	D	4.1	9.2	0.31
QTL21	GFD	Control	1	5A2	137.8	wsnp_Ex_rep_c101757_87064771	W	5.3	10.5	0.56
QTL21	SGW	Control	2	5A2	116.6	wsnp_Ku_c14139_22353229	D	4.1	7.9	0.98
QTL21	HI	Control	2	5A2	126.9	wsnp_Ex_c3838_6981043	D	5.4	6.7	0.72
QTL21	FW	Control (pre-heat)	2	5A2	131.2	wsnp_Ku_c23772_33711538	W	5.7	9.1	0.03
QTL21	GFD	HSI	2	5A2	134.1	wsnp_Ex_rep_c68829_67704044	D	4.8	11.6	0.14
QTL22	DTM	HSI	1	5A2	216.2	wsnp_Ex_c905_1748920	W	4.1	10.3	0.16
QTL23	SGW	Control	2	5B2	20.8	wsnp_Ku_c10296_17072695	D	5.7	7.9	0.98
QTL23	SGW	Heat	2	5B2	20.8	wsnp_Ku_c10296_17072695	D	4.4	7.1	0.99
QTL24	FW	Control (pre-heat)	1	5B2	62.5	wsnp_BE499835B_Ta_2_5	D	4.6	7.2	0.02
QTL24	FW	Control (pre-heat)	2	5B2	62.5	wsnp_BE499835B_Ta_2_5	D	6.9	11.3	0.03
QTL25	GFD	Control	2	6A	70.7	wsnp_JD_rep_c62949_40140212	D	7.0	6.9	0.50
QTL25	SGW	Control	1	6A	66.0	wsnp_Ex_c1104_2118684	D	4.2	5.5	1.06
QTL25	SGW	Control	2	6A	66.0	wsnp_Ex_c1104_2118684	D	7.1	9.8	1.09
QTL25	SGW	Heat	2	6A	66.0	wsnp_Ex_c1104_2118684	D	3.3	5.0	0.83
QTL26	GWS	Heat	2	6B2	27.3	wsnp_Ex_c42372_48966781	D	3.7	8.7	0.15
QTL26	GNS	Heat	1	6B2	27.3	wsnp_Ex_c42372_48966781	D	4.5	4.7	0.99
QTL26	ShW	Heat	2	6B2	27.3	wsnp_Ex_c42372_48966781	D	6.0	4.5	0.11
QTL27	AUSC	Heat	2	6B3	9.1	wsnp_Ex_c9038_15058444/ wsnp_Ex_c11573_18650189	D	3.7	9.1	19.53
QTL27	ChlR13	Heat	2	6B3	18.1	wsnp_Ex_c11573_18650189	D	4.8	8.9	0.21
QTL27	SGW	HSI	2	6B3	9.1	wsnp_Ex_c11573_18650189	W	3.8	12.1	0.17
QTL27	ChlR13	HSI	2	6B3	18.1	wsnp_Ex_c11573_18650189	W	4.8	8.9	0.46
QTL28	HI	Control	2	7A2	33.7	wsnp_Ex_c2268_4251636	W	3.6	5.5	0.65

Table S5 (continued)

QTL	Trait	Condition	Expt.	Linkage group	cM position	Most closely-associated marker(s)	Positive allele	Test statistic $-Log_{10}(p)$	R^2	Additive effect
QTL29	DTM	Control	1	7B	34.9	wsnp_Ex_c24376_33619527	D	10.6	29.6	1.60
QTL29	DTM	Control	2	7B	0.0	wsnp_CAP8_c334_304253	D	4.0	8.2	0.82
QTL29	GFD	Control	1	7B	34.9	wsnp_Ex_c24376_33619527	D	6.0	18.0	0.73
QTL29	GFD	Control	2	7B	0.0	wsnp_CAP8_c334_304253	D	18.0	18.2	0.82
QTL29	FLSe	Control	1	7B	25.4	wsnp_JD_c1285_1848292/ wsnp_Ex_c24376_33619527	D	8.1	26.5	3.53
QTL29	ShW	Control	1	7B	0.0	wsnp_CAP8_c334_304253	D	4.1	2.9	0.06
QTL29	DTA	Control (pre-heat)	1	7B	34.9	wsnp_Ex_c24376_33619527	D	6.1	14.5	0.92
QTL29	FL	Control (pre-heat)	1	7B	34.9	wsnp_Ex_c24376_33619527	D	5.3	16.9	0.45
QTL29	FL	Control (pre-heat)	2	7B	0.0	wsnp_CAP8_c334_304253	D	4.5	8.6	0.35
QTL29	DTM	Heat	1	7B	0.0	wsnp_CAP8_c334_304253	D	6.3	12.7	0.92
QTL29	DTM	Heat	2	7B	0.0	wsnp_CAP8_c334_304253	D	5.2	8.1	0.73
QTL29	GFD	Heat	1	7B	0.0	wsnp_CAP8_c334_304253	D	5.9	11.3	0.70
QTL29	GFD	Heat	2	7B	0.0	wsnp_CAP8_c334_304253	D	6.0	8.2	0.69
QTL29	ShW	Heat	1	7B	0.0	wsnp_CAP8_c334_304254	D	4.0	2.6	0.06
QTL29	HI	Heat	1	7B	6.5	wsnp_JD_c1285_1848292	W	4.4	9.9	0.32
QTL29	FLSe	HSI	1	7B	45.8	wsnp_Ex_rep_c68815_67687712	D	3.9	7.5	0.20

Positive allele: D, Drysdale; W, Waagan; Positive allele for Heat Susceptibility Index (HSI) means associated with intolerance.

Additive effect always refers to the effect of the positive allele.

DTA, days from sowing to anthesis; DTM, days from sowing to maturity defined as 95% spike senescence; GFD, grain-filling duration defined as days from anthesis to 95% spike senescence; FLSe, days from anthesis to 95% flag leaf senescence; GWS, grain weight spike^{-1} (g); GNS, grain number spike^{-1}; SGW, single grain weight (mg); ShW, shoot dry weight (g); PH, plant height (cm); ChlC10DAA, chlorophyll content 10 days after anthesis, i.e., just before heat treatment period (SPAD units); ChlC13DAA, chlorophyll content 13 days after anthesis, i.e., just after heat treatment period (SPAD units); AUSC, area under the SPAD curve made from measurements at 10, 13 and 27 days after anthesis, i.e., incorporates the period during-heat treatment and 2-weeks after; ChlR13, rate of chlorophyll change between 10 and 13 days after anthesis, i.e., during the heat treatment period (SPAD units day^{-1}); ChlR27, rate of chlorophyll change based on the linear regression of the measurements, at 10, 13 and 27 days after anthesis (SPAD units day^{-1}); FL, flag leaf length (cm); FW, flag leaf width (cm); HI, harvest index (%).

QTL16 may belong to QTL17 (Rht-B1) based on its effect.
