# Supplementary material for: A QTL on the short arm of wheat (Triticum aestivum L.) chromosome 3B affects the stability of grain weight in plants exposed to a brief heat shock early in grain filling
Source: BMC Plant Biol. 2016 Apr 22;16:100. doi: 10.1186/s12870-016-0784-6 (PMC4841048; doi:10.1186/s12870-016-0784-6)
Supplement: Additional file 6: Table S6. — Details of all QTL effects identified in the Drysdale × Waagan mapping population in the two heat tolerance experiments, with QTL effects arranged by trait/condition, then ordered by R2. (PDF 174 kb) [file 12870_2016_784_MOESM6_ESM.pdf]

Table S6. Details of all QTL effects identified in the Drysdale x Waagan mapping population in the two heat tolerance experiments, with QTL effects arranged by trait/condition, then ordered by R^2.

QTL	Trait	Condition	Expt.	Linkage group	cM position	Most closely-associated marker(s)	Positive allele	Test statistic $-Log_{10}(p)$	R^2	Additive effect
QTL18	DTA	Control (pre-heat)	1	4B	135.5	wsnp_Ex_c4148_7495656	W	21.7	37.1	1.48
QTL18	DTA	Control (pre-heat)	2	4B	135.5	wsnp_Ex_c4148_7495656	W	20.5	35.9	1.65
QTL29	DTA	Control (pre-heat)	1	7B	34.9	wsnp_Ex_c24376_33619527	D	6.1	14.5	0.92
QTL6	DTA	Control (pre-heat)	2	2B1	5.4	wsnp_Ra_c14112_22155451	D	3.8	6.9	0.72
QTL29	DTM	Control	1	7B	34.9	wsnp_Ex_c24376_33619527	D	10.6	29.6	1.60
QTL18	DTM	Control	2	4B	135.5	wsnp_Ex_c4148_7495656	W	8.8	19.2	1.25
QTL16	DTM	Control	1	4B	29.6	wsnp_Ex_c17561_26284693	W	5.0	10.7	0.96
QTL29	DTM	Control	2	7B	0.0	wsnp_CAP8_c334_304253	D	4.0	8.2	0.82
QTL18	DTM	Control	1	4B	135.5	wsnp_Ex_c4148_7495656	W	4.6	7.9	0.83
QTL18	DTM	Heat	2	4B	135.5	wsnp_Ex_c4148_7495656	W	12.8	22.2	1.21
QTL18	DTM	Heat	1	4B	141.3	wsnp_BE403378B_Ta_2_1	W	7.1	14.0	0.97
QTL29	DTM	Heat	1	7B	0.0	wsnp_CAP8_c334_304253	D	6.3	12.7	0.92
QTL19 (Rht-D1)	DTM	Heat	2	4D	0.0	Rht-D1	D	5.4	8.2	0.73
QTL29	DTM	Heat	2	7B	0.0	wsnp_CAP8_c334_304253	D	5.2	8.1	0.73
QTL5	DTM	Heat	1	2A	88.4	wsnp_Ex_c5984_10493714	W	3.8	7.1	0.69
QTL17 (Rht-B1)	DTM	Heat	2	4B	83.9	Rht-B1	W	3.8	5.9	0.62
QTL22	DTM	HSI	1	5A2	216.2	wsnp_Ex_c905_1748920	W	4.1	10.3	0.16
QTL29	GFD	Control	2	7B	0.0	wsnp_CAP8_c334_304253	D	18.0	18.2	0.82
QTL29	GFD	Control	1	7B	34.9	wsnp_Ex_c24376_33619527	D	6.0	18.0	0.73
QTL17 (Rht-B1)	GFD	Control	2	4B	86.3	wsnp_RFL_Contig4151_4728831	W	14.9	17.1	0.79
QTL19 (Rht-D1)	GFD	Control	2	4D	0.0	Rht-D1	D	10.8	12.1	0.66
QTL21	GFD	Control	1	5A2	137.8	wsnp_Ex_rep_c101757_87064771	W	5.3	10.5	0.56
QTL13	GFD	Control	1	4A1	0.0	wsnp_Ex_c11474_18507872	D	4.7	9.4	0.53
QTL18	GFD	Control	2	4B	135.5	wsnp_Ex_c4148_7495656	D	9.4	8.7	0.57
QTL1	GFD	Control	2	1A1	59.7	wsnp_Ex_c1997_3756118	D	6.3	7.3	0.52
QTL25	GFD	Control	2	6A	70.7	wsnp_JD_rep_c62949_40140212	D	7.0	6.9	0.50
QTL17 (Rht-B1)	GFD	Heat	2	4B	83.9	Rht-B1	W	11.1	21.2	1.11
QTL18	GFD	Heat	1	4B	108.3	wsnp_CAP12_rep_c4278_1949864	D	7.5	19.8	0.93
QTL19 (Rht-D1)	GFD	Heat	2	4D	0.0	Rht-D1	D	10.1	14.7	0.92
QTL11	GFD	Heat	1	3B1	1.4	wsnp_Ex_c12875_20407926	W	6.4	13.1	0.76
QTL18	GFD	Heat	2	4B	127.5	wsnp_Ku_c11570_18860306	D	5.4	12.9	0.87
QTL29	GFD	Heat	1	7B	0.0	wsnp_CAP8_c334_304253	D	5.9	11.3	0.70
QTL29	GFD	Heat	2	7B	0.0	wsnp_CAP8_c334_304253	D	6.0	8.2	0.69
QTL15	GFD	Heat	2	4A2	42.3	wsnp_RFL_Contig25_2082245	D	4.6	4.2	0.49
QTL18	GFD	HSI	1	4B	99.5	wsnp_CAP12_rep_c4278_1949864	W	5.5	12.4	0.13
QTL21	GFD	HSI	2	5A2	134.1	wsnp_Ex_rep_c68829_67704044	D	4.8	11.6	0.14
QTL11	GFD	HSI	1	3B1	3.2	wsnp_BE497169B_Ta_2_1	D	4.4	10.0	0.12
QTL15	GFD	HSI	2	4A2	42.3	wsnp_RFL_Contig25_2082245	W	3.8	8.4	0.12
QTL18	FLSe	Control	2	4B	135.5	wsnp_Ex_c4148_7495656	D	16.4	27.9	2.02
QTL29	FLSe	Control	1	7B	25.4	wsnp_JD_c1285_1848292/ wsnp_Ex_c24376_33619527	D	8.1	26.5	3.53
QTL19 (Rht-D1)	FLSe	Control	2	4D	0.0	Rht-D1	D	12.5	19.1	1.68
QTL17 (Rht-B1)	FLSe	Control	1	4B	83.9	Rht-B1	W	6.6	12.2	2.40
QTL17 (Rht-B1)	FLSe	Control	2	4B	86.3	wsnp_RFL_Contig4151_4728831	W	7.4	11.9	1.32
QTL19 (Rht-D1)	FLSe	Control	1	4D	0.0	Rht-D1	D	6.2	11.3	2.30
QTL1	FLSe	Control	2	1A1	56.1	wsnp_Ku_c10292_17066821	D	3.8	5.4	0.89
QTL18	FLSe	Heat	2	4B	149.9	wsnp_BE403378B_Ta_2_1/ wsnp_CAP7_c5487_2464794	D	5.7	14.8	2.15
QTL11	FLSe	Heat	1	3B1	3.2	wsnp_BE497169B_Ta_2_1	W	5.5	14.2	2.06
QTL19 (Rht-D1)	FLSe	Heat	2	4D	0.0	Rht-D1	D	5.5	11.6	1.90
QTL18	FLSe	HSI	1	4B	117.2	wsnp_Ex_c39876_47057394	W	6.5	17.7	0.30
QTL11	FLSe	HSI	1	3B1	1.4	wsnp_Ex_c12875_20407926	D	6.7	14.4	0.27
QTL29	FLSe	HSI	1	7B	45.8	wsnp_Ex_rep_c68815_67687712	D	3.9	7.5	0.20

Table S6 (continued).

QTL	Trait	Condition	Expt.	Linkage group	cM position	Most closely-associated marker(s)	Positive allele	Test statistic $-Log_{10}(p)$	R^2	Additive effect
QTL19 (Rht-D1)	GWS	Control	1	4D	0.0	Rht-D1	W	29.6	37.0	0.19
QTL19 (Rht-D1)	GWS	Control	2	4D	0.0	Rht-D1	W	8.4	15.4	0.26
QTL17 (Rht-B1)	GWS	Control	1	4B	83.9	Rht-B1	D	12.0	15.2	0.12
QTL18	GWS	Control	2	4B	135.5	wsnp_Ex_c4148_7495656	W	7.1	12.5	0.23
QTL18	GWS	Control	1	4B	135.5	wsnp_Ex_c4148_7495656	W	7.8	9.5	0.09
QTL9	GWS	Control	1	2D4	25.6	wsnp_Ku_c30494_40319867	D	5.4	7.9	0.09
QTL2	GWS	Control	2	1A2	0.0	wsnp_Ku_c40759_48907151	D	3.9	6.7	0.17
QTL19 (Rht-D1)	GWS	Heat	1	4D	0.0	Rht-D1	W	23.8	28.7	0.18
QTL19 (Rht-D1)	GWS	Heat	2	4D	0.0	Rht-D1	W	12.4	23.1	0.25
QTL18	GWS	Heat	2	4B	149.9	wsnp_BE403378B_Ta_2_1	W	7.1	15.6	0.20
QTL17 (Rht-B1)	GWS	Heat	1	4B	83.9	Rht-B1	D	12.5	15.1	0.13
QTL11	GWS	Heat	1	3B1	11.0	wsnp_BE497169B_Ta_2_1	W	7.1	11.2	0.11
QTL26	GWS	Heat	2	6B2	27.3	wsnp_Ex_c42372_48966781	D	3.7	8.7	0.15
QTL9	GWS	Heat	1	2D4	25.6	wsnp_Ku_c30494_40319867	D	5.5	7.8	0.09
QTL17 (Rht-B1)	GWS	Heat	2	4B	83.9	Rht-B1	D	3.8	5.7	0.12
QTL18	GWS	Heat	1	4B	135.5	wsnp_Ex_c4148_7495656	W	4.1	4.4	0.07
QTL11	GWS	HSI	1	3B1	3.2	wsnp_BE497169B_Ta_2_1	D	8.8	21.6	1.16
QTL11	GWS	HSI	2	3B1	0.0	wsnp_Ra_c41135_48426638	D	5.9	15.0	0.38
QTL19 (Rht-D1)	GNS	Control	1	4D	0.0	Rht-D1	W	10.7	21.4	2.13
QTL18	GNS	Control	2	4B	135.5	wsnp_Ex_c4148_7495656	W	9.0	15.5	3.83
QTL19 (Rht-D1)	GNS	Control	2	4D	0.0	Rht-D1	W	7.5	13.1	3.53
QTL18	GNS	Control	1	4B	135.5	wsnp_Ex_c4148_7495656	W	5.5	10.3	1.48
QTL2	GNS	Control	2	1A2	0.0	wsnp_Ku_c40759_48907151	D	4.7	7.9	2.73
QTL19 (Rht-D1)	GNS	Heat	2	4D	0.0	Rht-D1	W	10.3	18.9	3.86
QTL18	GNS	Heat	2	4B	135.5	wsnp_Ex_c4148_7495656	W	9.6	17.5	3.71
QTL19 (Rht-D1)	GNS	Heat	1	4D	8.0	wsnp_Ex_rep_c107564_91144523	W	8.2	16.5	1.85
QTL18	GNS	Heat	1	4B	135.5	wsnp_Ex_c4148_7495656	W	3.9	4.7	0.98
QTL26	GNS	Heat	1	6B2	27.3	wsnp_Ex_c42372_48966781	D	4.5	4.7	0.99
QTL17 (Rht-B1)	SGW	Control	1	4B	83.9	Rht-B1	D	14.2	21.5	2.09
QTL17 (Rht-B1)	SGW	Control	2	4B	83.9	Rht-B1	D	10.5	15.8	1.38
QTL19 (Rht-D1)	SGW	Control	2	4D	0.0	Rht-D1	W	9.1	12.9	1.25
QTL19 (Rht-D1)	SGW	Control	1	4D	0.0	Rht-D1	W	8.0	11.5	1.27
QTL25	SGW	Control	2	6A	66.0	wsnp_Ex_c1104_2118684	D	7.1	9.8	1.09
QTL21	SGW	Control	2	5A2	116.6	wsnp_Ku_c14139_22353229	D	4.1	7.9	0.98
QTL23	SGW	Control	2	5B2	20.8	wsnp_Ku_c10296_17072695	D	5.7	7.9	0.98
QTL9	SGW	Control	2	2D4	32.4	wsnp_RFL_Contig2659_2346243	D	3.5	6.1	0.86
QTL1	SGW	Control	1	1A1	36.5	wsnp_Ex_c200_391493	W	4.3	5.6	1.07
QTL25	SGW	Control	1	6A	66.0	wsnp_Ex_c1104_2118684	D	4.2	5.5	1.06
QTL17 (Rht-B1)	SGW	Heat	1	4B	86.3	wsnp_RFL_Contig4151_4728831	D	26.0	33.0	2.81
QTL11	SGW	Heat	1	3B1	11.0	wsnp_BE497169B_Ta_2_1	W	7.0	11.5	1.65
QTL17 (Rht-B1)	SGW	Heat	2	4B	83.9	Rht-B1	D	6.1	10.2	1.19
QTL19 (Rht-D1)	SGW	Heat	1	4D	0.0	Rht-D1	W	7.6	10.0	1.17
QTL19 (Rht-D1)	SGW	Heat	2	4D	0.0	Rht-D1	W	5.8	9.5	1.15
QTL9	SGW	Heat	2	2D4	32.4	wsnp_RFL_Contig2659_2346243	D	3.5	7.5	1.02
QTL23	SGW	Heat	2	5B2	20.8	wsnp_Ku_c10296_17072695	D	4.4	7.1	0.99
QTL3	SGW	Heat	2	1B	83.4	wsnp_Ku_c18227_27490539	W	4.3	6.8	0.97
QTL12	SGW	Heat	2	3B2	54.9	wsnp_Ex_c1097_2105209	W	3.8	5.9	0.91
QTL14	SGW	Heat	1	4A2	0.0	wsnp_Ex_c41074_47987860	W	4.7	5.1	1.11
QTL25	SGW	Heat	2	6A	66.0	wsnp_Ex_c1104_2118684	D	3.3	5.0	0.83
QTL11	SGW	HSI	1	3B1	3.2	wsnp_BE497169B_Ta_2_1	D	8.1	20.1	0.92
QTL27	SGW	HSI	2	6B3	9.1	wsnp_Ex_c11573_18650189	W	3.8	12.1	0.17
QTL11	SGW	HSI	2	3B1	1.4	wsnp_Ex_c12875_20407926	D	4.7	10.8	0.16

Table S6 (continued).

QTL	Trait	Condition	Expt.	Linkage group	cM position	Most closely-associated marker(s)	Positive allele	Test statistic $-Log_{10}(p)$	R^2	Additive effect
QTL19 (Rht-D1)	ShW	Control	2	4D	0.0	Rht-D1	W	50.5	48.7	0.37
QTL19 (Rht-D1)	ShW	Control	1	4D	0.0	Rht-D1	W	39.9	47.3	0.23
QTL17 (Rht-B1)	ShW	Control	2	4B	83.9	Rht-B1	D	19.6	20.7	0.24
QTL17 (Rht-B1)	ShW	Control	1	4B	83.9	Rht-B1	D	16.8	18.6	0.14
QTL18	ShW	Control	2	4B	135.5	wsnp_Ex_c4148_7495658	W	11.1	9.8	0.17
QTL9	ShW	Control	1	2D4	25.6	wsnp_Ku_c30494_40319867	D	4.0	5.6	0.08
QTL18	ShW	Control	1	4B	135.5	wsnp_Ex_c4148_7495656	W	4.4	5.1	0.07
QTL29	ShW	Control	1	7B	0.0	wsnp_CAP8_c334_304253	D	4.1	2.9	0.06
QTL9	ShW	Control	2	2D4	18.8	wsnp_Ku_c30494_40319867	D	4.0	2.8	0.09
QTL19 (Rht-D1)	ShW	Heat	2	4D	0.0	Rht-D1	W	55.0	42.5	0.35
QTL19 (Rht-D1)	ShW	Heat	1	4D	0.0	Rht-D1	W	58.9	37.9	0.22
QTL17 (Rht-B1)	ShW	Heat	2	4B	83.9	Rht-B1	D	32.6	26.5	0.28
QTL17 (Rht-B1)	ShW	Heat	1	4B	83.9	Rht-B1	D	34.2	23.1	0.17
QTL11	ShW	Heat	1	3B1	11.0	wsnp_BE497169B_Ta_2_1	W	17.9	14.2	0.14
QTL18	ShW	Heat	2	4B	135.5	wsnp_Ex_c4148_7495659	W	12.7	10.3	0.17
QTL26	ShW	Heat	2	6B2	27.3	wsnp_Ex_c42372_48966781	D	6.0	4.5	0.11
QTL9	ShW	Heat	1	2D4	25.6	wsnp_Ku_c30494_40319867	D	5.4	4.3	0.07
QTL11	ShW	Heat	2	3B1	40.5	wsnp_Ku_c3817_7009093/ wsnp_Ex_c44375_50444756	W	5.8	3.5	0.10
QTL8	ShW	Heat	2	2D3	3.6	wsnp_JD_c5919_7081809	D	4.6	3.2	0.10
QTL18	ShW	Heat	1	4B	135.5	wsnp_Ex_c4148_7495657	W	5.0	2.6	0.06
QTL29	ShW	Heat	1	7B	0.0	wsnp_CAP8_c334_304254	D	4.0	2.6	0.06
QTL1	ShW	Heat	2	1A1	48.2	wsnp_Ku_c23926_33870364	W	4.9	2.0	0.08
QTL10	ShW	Heat	1	3A2	15.3	wsnp_Ex_c4069_7354375	D	3.9	2.0	0.05
QTL14	ShW	Heat	1	4A2	0.0	wsnp_Ex_c41074_47987860	W	3.8	1.4	0.04
QTL11	ShW	HSI	1	3B1	3.2	wsnp_BE497169B_Ta_2_1	D	9.3	22.8	1.62
QTL19 (Rht-D1)	PH	Control	1	4D	0.0	Rht-D1	W	50.0	56.5	13.87
QTL19 (Rht-D1)	PH	Control	2	4D	0.0	Rht-D1	W	62.3	56.4	14.11
QTL17 (Rht-B1)	PH	Control	2	4B	83.9	Rht-B1	D	83.9	37.1	11.44
QTL17 (Rht-B1)	PH	Control	1	4B	83.9	Rht-B1	D	37.6	32.9	10.58
QTL15	PH	Control	1	4A2	41.6	wsnp_Ex_rep_c68569_67411985	W	5.6	2.2	2.74
QTL15	PH	Control	2	4A2	42.3	wsnp_RFL_Contig25_2082245	W	9.0	1.8	2.52
QTL10	PH	Control	2	3A2	15.3	wsnp_Ex_c4069_7354375	D	7.1	1.5	2.27
QTL1	PH	Control	2	1A1	66.2	wsnp_BE517729A_Ta_2_1	W	4.2	1.1	1.94
QTL19 (Rht-D1)	PH	Heat	1	4D	0.0	Rht-D1	W	56.5	56.4	13.86
QTL19 (Rht-D1)	PH	Heat	2	4D	0.0	Rht-D1	W	54.0	52.6	13.78
QTL17 (Rht-B1)	PH	Heat	1	4B	83.9	Rht-B1	D	46.3	36.8	11.19
QTL17 (Rht-B1)	PH	Heat	2	4B	83.9	Rht-B1	D	44.9	35.9	11.38
QTL15	PH	Heat	2	4A2	42.3	wsnp_RFL_Contig25_2082245	W	7.3	2.3	2.90
QTL10	PH	Heat	2	3A2	15.3	wsnp_Ex_c4069_7354376	D	6.6	2.1	2.75
QTL15	PH	Heat	1	4A2	41.6	wsnp_Ex_rep_c68569_67411985	W	6.0	1.8	2.47
QTL1	PH	Heat	2	1A1	67.6	wsnp_Ex_c5060_8985678	W	4.9	1.5	2.31
QTL5	PH	Heat	1	2A	60.1	wsnp_BQ168780B_Ta_2_1	D	4.3	1.3	2.08
QTL10	PH	Heat	1	3A2	24.0	wsnp_Ex_c1141_2191485	D	3.9	1.1	1.94

Table S6 (continued).

QTL	Trait	Condition	Expt.	Linkage group	cM position	Most closely-associated marker(s)	Positive allele	Test statistic -Log$_{10}$(p)	R^2	Additive effect
QTL11	ChlC10DAA	Control (pre-heat)	1	3B1	1.4	wsnp_Ex_c12875_20407926	W	10.7	17.9	0.71
QTL11	ChlC10DAA	Control (pre-heat)	2	3B1	1.4	wsnp_Ex_c12875_20407926	W	7.4	16.8	0.65
QTL8	ChlC10DAA	Control (pre-heat)	1	2D3	0.0	wsnp_Ex_c2258_4232538	D	8.3	14.0	0.63
QTL20	ChlC10DAA	Control (pre-heat)	1	5A2	3.8	wsnp_Ex_c1481_2831499	D	6.3	12.1	0.59
QTL5	ChlC10DAA	Control (pre-heat)	2	2A	108.4	wsnp_Ex_c3808_6924802	W	4.3	8.7	0.47
QTL2	ChlC10DAA	Control (pre-heat)	1	1A2	0.0	wsnp_Ku_c40759_48907151	D	3.8	4.1	0.34
QTL5	ChlC10DAA	Control (pre-heat)	1	2A	75.0	wsnp_Ex_c42720_49228237	W	4.0	4.1	0.34
QTL11	ChlC13DAA	Control	2	3B1	1.4	wsnp_Ex_c12875_20407926	W	8.9	19.4	0.67
QTL11	ChlC13DAA	Control	1	3B1	1.4	wsnp_Ex_c12875_20407926	W	7.7	14.6	0.59
QTL8	ChlC13DAA	Control	1	2D3	0.0	wsnp_Ex_c2258_4232538	D	8.3	12.7	0.55
QTL5	ChlC13DAA	Control	2	2A	78.0	wsnp_Ex_rep_c102538_87682273	W	5.1	10.0	0.49
QTL20	ChlC13DAA	Control	1	5A2	11.9	wsnp_Ex_c1481_2831499	D	6.7	8.6	0.45
QTL11	ChlC13DAA	Heat	1	3B1	1.4	wsnp_Ex_c12875_20407926	W	26.0	42.0	2.01
QTL11	ChlC13DAA	Heat	2	3B1	0.0	wsnp_Ra_c41135_48426638	W	16.0	34.3	1.95
QTL8	ChlC13DAA	Heat	1	2D3	5.8	wsnp_Ex_c7260_12463738	D	6.1	8.5	0.91
QTL20	ChlC13DAA	Heat	1	5A2	1.5	wsnp_JD_c43389_30288993	D	4.6	6.3	0.78
QTL11	ChlC13DAA	HSI	1	3B1	1.4	wsnp_Ex_c12875_20407926	D	16.6	35.7	0.67
QTL11	ChlC13DAA	HSI	2	3B1	0.0	wsnp_Ra_c41135_48426638	D	10.0	23.9	0.81
QTL11	ChlC27DAA	Control	2	3B1	0.0	wsnp_Ra_c41135_48426638	W	11.5	23.0	0.68
QTL11	ChlC27DAA	Control	1	3B1	0.0	wsnp_Ra_c41135_48426638	W	8.6	17.1	0.66
QTL8	ChlC27DAA	Control	1	2D3	0.0	wsnp_Ex_c2258_4232538	D	6.4	11.6	0.55
QTL6	ChlC27DAA	Control	2	2B1	0.0	wsnp_Ra_c14112_22155451	W	6.0	11.5	0.48
QTL5	ChlC27DAA	Control	2	2A	80.9	wsnp_Ra_c4503_8155485	W	5.2	9.2	0.43
QTL5	ChlC27DAA	Control	1	2A	75.0	wsnp_Ex_c42720_49228237	W	5.0	9.0	0.48
QTL19 (Rht-D1)	ChlC27DAA	Control	2	4D	2.9	wsnp_CAP11_c356_280910	D	3.7	6.3	0.36
QTL11	ChlC27DAA	Heat	1	3B1	1.4	wsnp_Ex_c12875_20407926	W	36.3	54.4	2.49
QTL11	ChlC27DAA	Heat	2	3B1	0.0	wsnp_Ra_c41135_48426638	W	8.0	19.9	2.55
QTL8	ChlC27DAA	Heat	1	2D3	5.0	wsnp_Ex_c20011_29041563	D	5.4	6.7	0.87
QTL11	ChlC27DAA	HSI	1	3B1	1.4	wsnp_Ex_c12875_20407926	D	21.3	38.6	1.28
QTL11	ChlC27DAA	HSI	2	3B1	0.0	wsnp_Ra_c41135_48426638	D	5.3	13.4	0.62
QTL13	ChlC27DAA	HSI	1	4A1	0.0	wsnp_Ex_c11474_18507872	D	4.7	7.4	0.56
QTL11	AUSC	Control	2	3B1	1.4	wsnp_Ex_c12875_20407926	W	10.5	20.4	10.98
QTL11	AUSC	Control	1	3B1	0.0	wsnp_Ra_c41135_48426638	W	7.5	15.2	10.78
QTL8	AUSC	Control	1	2D3	0.0	wsnp_Ex_c2258_4232538	D	7.1	13.3	10.09
QTL5	AUSC	Control	2	2A	80.9	wsnp_Ra_c4503_8155485	W	5.9	10.5	7.86
QTL5	AUSC	Control	1	2A	75.0	wsnp_Ex_c42720_49228237	W	4.5	8.1	7.88
QTL6	AUSC	Control	2	2B1	0.0	wsnp_Ra_c14112_22155451	W	4.3	7.9	6.83
QTL11	AUSC	Heat	1	3B1	1.4	wsnp_Ex_c12875_20407926	W	33.2	48.6	36.94
QTL11	AUSC	Heat	2	3B1	0.0	wsnp_Ra_c41135_48426638	W	13.3	28.0	34.31
QTL27	AUSC	Heat	2	6B3	9.1	wsnp_Ex_c9038_15058444/ wsnp_Ex_c11573_18650189	D	3.7	9.1	19.53
QTL8	AUSC	Heat	1	2D3	5.8	wsnp_Ex_c7260_12463738	D	6.4	8.1	15.04
QTL20	AUSC	Heat	1	5A2	1.5	wsnp_JD_c43389_30288993	D	4.4	5.4	12.28
QTL11	AUSC	HSI	1	3B1	1.4	wsnp_Ex_c12875_20407926	D	20.5	38.3	0.91
QTL11	AUSC	HSI	2	3B1	0.0	wsnp_Ra_c41135_48426638	D	7.3	18.3	0.67
QTL13	AUSC	HSI	1	4A1	0.0	wsnp_Ex_c11474_18507872	D	3.8	6.1	0.36
QTL11	ChlR13	Heat	1	3B1	0.0	wsnp_Ra_c41135_48426638	W	19.7	39.6	0.37
QTL11	ChlR13	Heat	2	3B1	0.0	wsnp_Ra_c41135_48426638	W	13.2	27.2	0.37
QTL27	ChlR13	Heat	2	6B3	18.1	wsnp_Ex_c11573_18650189	D	4.8	8.9	0.21
QTL11	ChlR13	HSI	1	3B1	0.0	wsnp_Ra_c41135_48426638	D	16.3	39.7	0.55
QTL11	ChlR13	HSI	2	3B1	0.0	wsnp_Ra_c41135_48426638	D	13.2	27.2	0.80
QTL27	ChlR13	HSI	2	6B3	18.1	wsnp_Ex_c11573_18650189	W	4.8	8.9	0.46
QTL2	ChlR27	Control	2	1A2	0.0	wsnp_Ku_c40759_48907151	W	4.6	11.3	0.04
QTL11	ChlR27	Heat	1	3B1	0.0	wsnp_Ra_c41135_48426638	W	29.5	50.4	0.07
QTL2	ChlR27	Heat	2	1A2	0.0	wsnp_Ku_c40759_48907151	W	4.4	10.7	0.10
QTL11	ChlR27	HSI	2	3B1	0.0	wsnp_Ra_c41135_48426638	D	9.3	19.4	0.40
QTL2	ChlR27	HSI	2	1A2	0.0	wsnp_Ku_c40759_48907151	D	5.9	11.0	0.30
QTL18	ChlR27	HSI	2	4B	135.5	wsnp_Ex_c4148_7495656	W	4.7	8.4	0.26

Table S6 (continued).

QTL	Trait	Condition	Expt.	Linkage group	cM position	Most closely-associated marker(s)	Positive allele	Test statistic $-Log_{10}(p)$	R^2	Additive effect
QTL29	FL	Control (pre-heat)	1	7B	34.9	wsnp_Ex_c24376_33619527	D	5.3	16.9	0.45
QTL7	FL	Control (pre-heat)	1	2B1	133.1	wsnp_RFL_Contig1892_1042675	D	6.4	13.9	0.41
QTL18	FL	Control (pre-heat)	2	4B	126.1	wsnp_Ex_c39876_47057394	D	6.9	13.6	0.44
QTL7	FL	Control (pre-heat)	2	2B1	126.3	wsnp_JD_c6010_7167159	D	6.2	13.5	0.43
QTL29	FL	Control (pre-heat)	2	7B	0.0	wsnp_CAP8_c334_304253	D	4.5	8.6	0.35
QTL4	FL	Control (pre-heat)	1	2A	0.0	wsnp_Ex_c2772_5130007	W	3.9	7.6	0.30
QTL18	FW	Control (pre-heat)	2	4B	117.2	wsnp_Ex_c39876_47057394	W	16.9	31.2	0.06
QTL18	FW	Control (pre-heat)	1	4B	108.3	wsnp_CAP12_rep_c4278_1949864	W	10.1	20.4	0.03
QTL24	FW	Control (pre-heat)	2	5B2	62.5	wsnp_BE499835B_Ta_2_5	D	6.9	11.3	0.03
QTL21	FW	Control (pre-heat)	2	5A2	131.2	wsnp_Ku_c23772_33711538	W	5.7	9.1	0.03
QTL24	FW	Control (pre-heat)	1	5B2	62.5	wsnp_BE499835B_Ta_2_5	D	4.6	7.2	0.02
QTL5	FW	Control (pre-heat)	2	2A	109.9	wsnp_Ex_c59095_60108185	W	5.4	6.8	0.03
QTL10	FW	Control (pre-heat)	2	3A2	30.4	wsnp_Ex_c25668_34932304	D	5.4	6.0	0.02
QTL15	FW	Control (pre-heat)	2	4A2	47.3	wsnp_Ex_c55245_57821389	D	4.7	6.0	0.02
QTL17 (Rht-B1)	HI	Control	2	4B	77.7	wsnp_Ex_c18433_27269748/ Rht-B1	W	15.8	23.8	1.35
QTL11	HI	Control	2	3B1	0.0	wsnp_Ra_c41135_48426638	D	8.7	11.5	0.94
QTL19 (Rht-D1)	HI	Control	2	4D	0.0	Rht-D1	D	8.4	10.6	0.90
QTL21	HI	Control	2	5A2	126.9	wsnp_Ex_c3838_6981043	D	5.4	6.7	0.72
QTL28	HI	Control	2	7A2	33.7	wsnp_Ex_c2268_4251636	W	3.6	5.5	0.65
QTL17 (Rht-B1)	HI	Heat	2	4B	77.7	wsnp_Ex_c18433_27269748/ Rht-B1	W	12.0	25.4	1.22
QTL19 (Rht-D1)	HI	Heat	2	4D	0.0	Rht-D1	D	10.8	19.4	1.07
QTL29	HI	Heat	1	7B	6.5	wsnp_JD_c1285_1848292	W	4.4	9.9	0.32
QTL20	HI	Heat	1	5A2	0.0	wsnp_CAP11_c923_558715	D	4.1	9.2	0.31
QTL11	HI	HSI	2	3B1	0.0	wsnp_Ra_c41135_48426638	D	4.1	10.3	0.29

Positive allele: D, Drysdale; W, Waagan; Positive allele for Heat Susceptibility Index (HSI) means associated with intolerance.

Additive effect always refers to the effect of the positive allele.

Degree of green shading indicates magnitude of LOD and R^2 values, to aid visualization.

DTA, days from sowing to anthesis; DTM, days from sowing to maturity defined as 95% spike senescence; GFD, grain-filling duration defined as days from anthesis to 95% spike senescence; FLSe, days from anthesis to 95% flag leaf senescence; GWS, grain weight spike^{-1} (g); GNS, grain number spike^{-1}; SGW, single grain weight (mg); ShW, shoot dry weight (g); PH, plant height (cm); ChlC10DAA, chlorophyll content 10 days after anthesis, i.e., just before heat treatment period (SPAD units); ChlC13DAA, chlorophyll content 13 days after anthesis, i.e., just after heat treatment period (SPAD units); AUSC, area under the SPAD curve made from measurements at 10, 13 and 27 days after anthesis, i.e., incorporates the period during-heat treatment and 2-weeks after; ChlR13, rate of chlorophyll change between 10 and 13 days after anthesis, i.e., during the heat treatment period (SPAD units day^{-1}); ChlR27, rate of chlorophyll change based on the linear regression of the measurements, at 10, 13 and 27 days after anthesis (SPAD units day^{-1}); FL, flag leaf length (cm); FW, flag leaf width (cm); HI, harvest index (%).

QTL16 may belong to QTL17 (Rht-B1) based on its effect.
