# Supplementary material for: A QTL on the short arm of wheat (Triticum aestivum L.) chromosome 3B affects the stability of grain weight in plants exposed to a brief heat shock early in grain filling
Source: BMC Plant Biol. 2016 Apr 22;16:100. doi: 10.1186/s12870-016-0784-6 (PMC4841048; doi:10.1186/s12870-016-0784-6)
Supplement: Additional file 8: Figure S1. — Molecular marker genetic map made from the Drysdale × Waagan population of 139 F1-derived doubled haploid lines. The map is represented here using a set of 551 genetically non-redundant markers; an (R) after a marker indicates genetic redundancy with other markers. Linkage groups were ordered and oriented along each chromosome so they aligned to the wheat consensus SNP map of Cavanagh et al. [34] and so the end of the short arm was at the top. The numbers to the left of each linkage group show cM distances from the top of each linkage group. Markers in bold were monomorphic in 2 to 8 of the 13 DH sub-populations (derived from different F1 plants) and may therefore identify chromosome segments that were heterogeneous within a parent variety. Markers that showed segregation distortion are in colour, with blue and red indicating an excess of Drysdale or Waagan alleles, respectively. The significance levels of segregation distortion are represent by asterisks (*p < 0.05, **p < 0.01 and ***p < 0.001). (PDF 119 kb) [file 12870_2016_784_MOESM8_ESM.pdf]

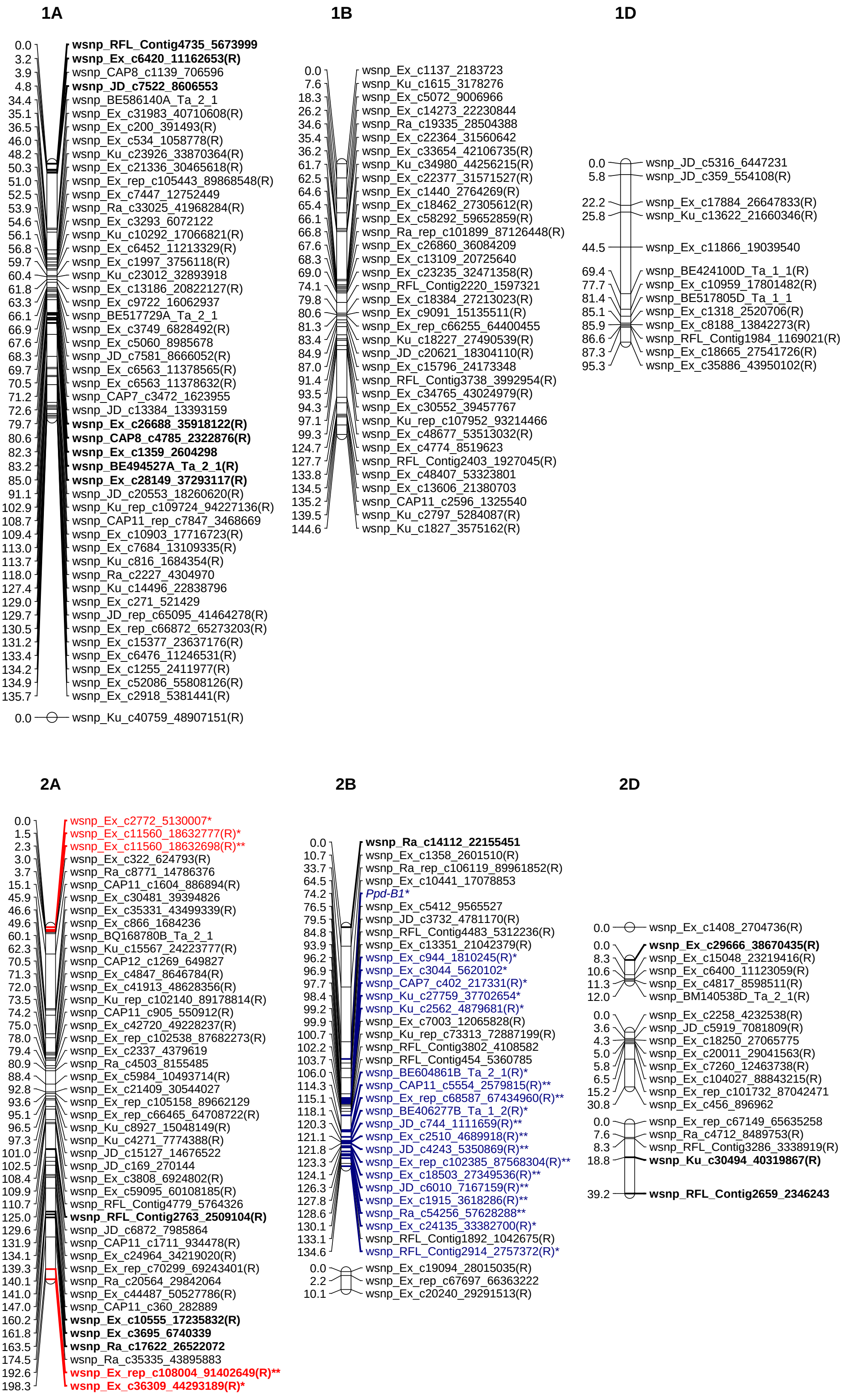

Figure S1. Molecular marker genetic map made from the Drysdale x Waagan population of 139 F_1-derived doubled haploid (DH) lines. The map is represented here using a set of 551 genetically non-redundant markers; an (R) after a marker indicates genetic redundancy with other markers. Linkage groups were ordered and oriented along each chromosome so they aligned to the wheat consensus SNP map of Cavanaugh et al. (2013) and so the end of the short arm was at the top. The numbers to the left of each linkage group show cM distances from the top of each linkage group. Markers in bold were monomorphic in 2 to 8 of the 13 DH sub-populations (derived from different F_1 plants) and may therefore identify chromosome segments that were heterogeneous within a parent variety. Markers that showed segregation distortion are in colour, with blue and red indicating an excess of Drysdale or Waagan alleles, respectively. The significance levels of segregation distortion are represent by asterisks (*$p < 0.05$, **$p < 0.01$ and ***$p < 0.001$).

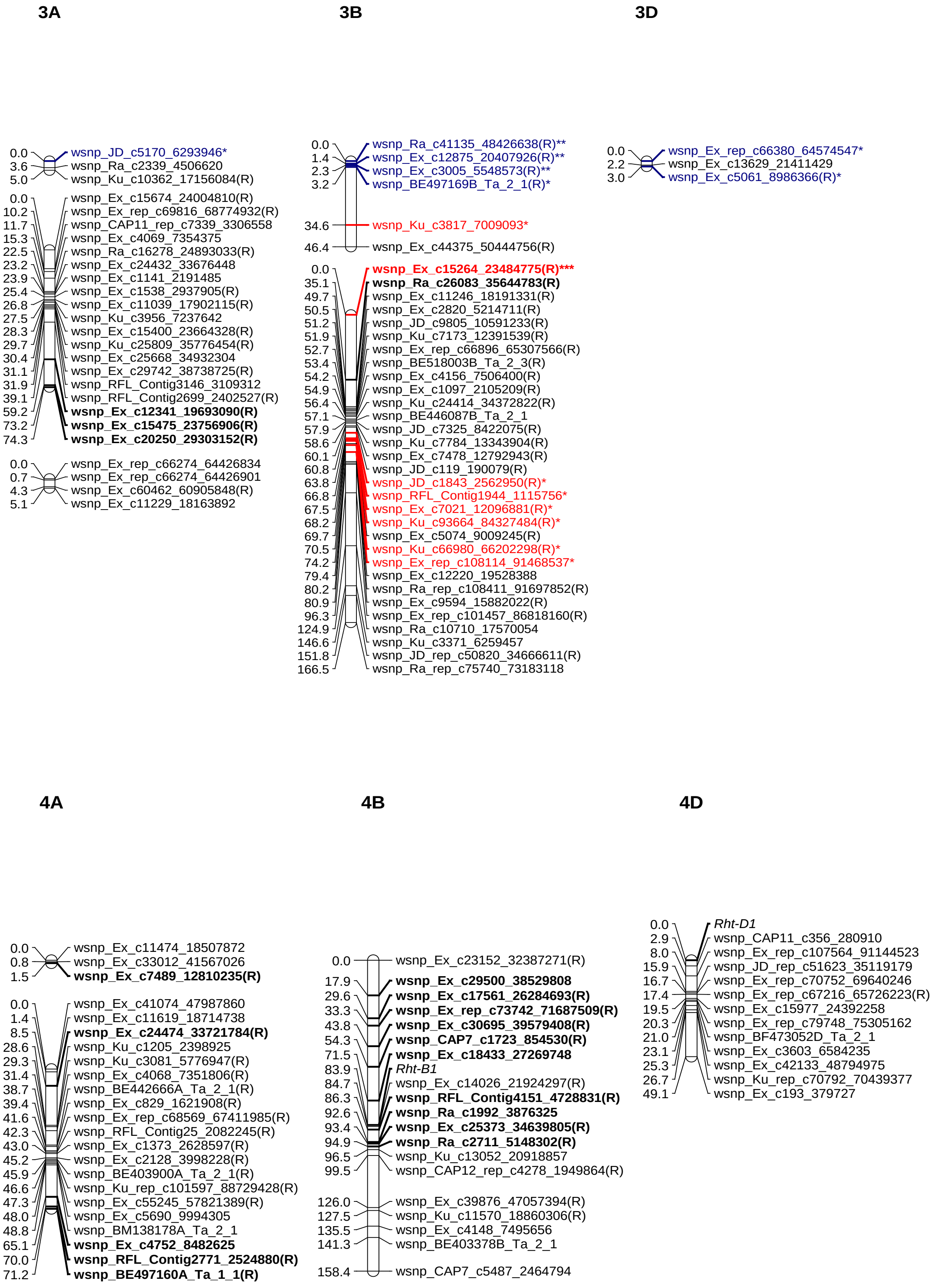

Figure S1 (continued)

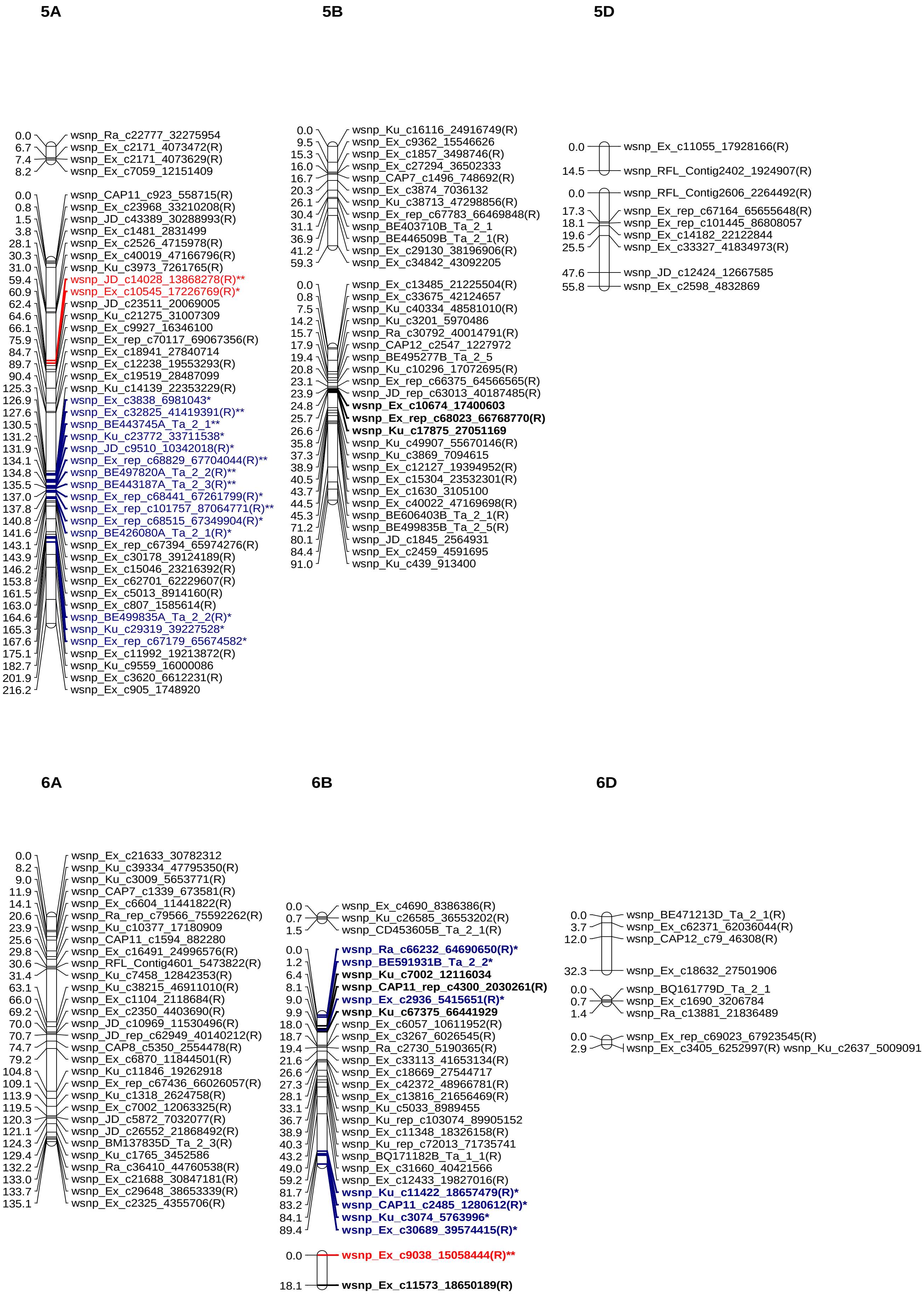

Figure S1 (continued)

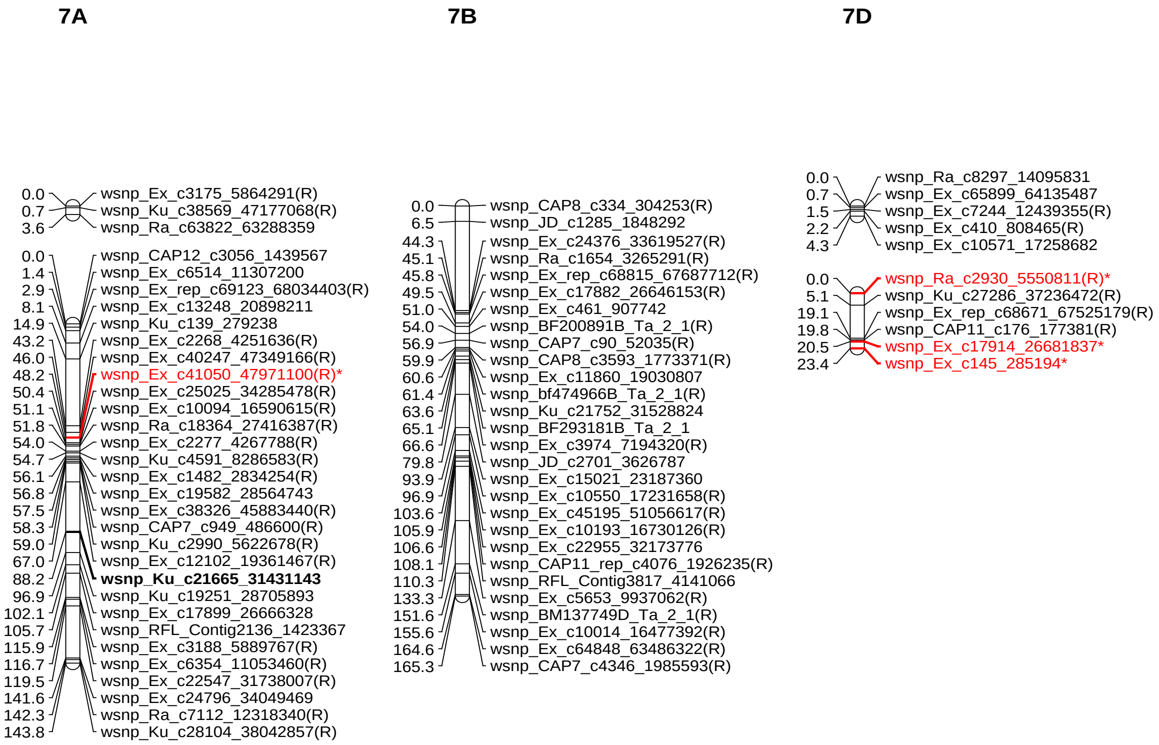

Figure S1 (continued)
