# Supplementary material for: A QTL on the short arm of wheat (Triticum aestivum L.) chromosome 3B affects the stability of grain weight in plants exposed to a brief heat shock early in grain filling
Source: BMC Plant Biol. 2016 Apr 22;16:100. doi: 10.1186/s12870-016-0784-6 (PMC4841048; doi:10.1186/s12870-016-0784-6)
Supplement: Additional file 9: Table S8. — Summary statistics, by chromosome, for the Drysdale × Waagan linkage map (PDF 73 kb) [file 12870_2016_784_MOESM9_ESM.pdf]

Table S8. Summary statistics, by chromosome, for the Drysdale x Waagan linkage map.

Chromosome	Number of non-redundant loci	Number of all loci	Length (cM)	Marker density* (cM per non-redundant locus)	Maximum spacing
**1A**	50	182	136	2.7	29.6
**1B**	35	127	145	4.1	25.6
**1D**	13	75	95	7.3	25.0
**2A**	45	267	198	4.4	30.8
**2B**	36	178	145	4.0	30.8
**2D**	19	56	82	4.3	20.4
**3A**	26	147	84	3.3	20.0
**3B**	37	183	213	5.8	35.1
**3D**	3	12	3	1.0	2.2
**4A**	23	129	73	3.2	20.0
**4B**	20	55	158	7.9	26.6
**4D**	13	18	49	3.8	22.4
**5A**	46	195	224	4.9	34.9
**5B**	36	160	150	4.2	25.9
**5D**	9	26	70	7.8	22.0
**6A**	30	230	135	4.5	31.7
**6B**	29	283	109	3.8	22.5
**6D**	10	27	37	3.7	20.3
**7A**	32	159	147	4.6	28.2
**7B**	28	163	165	5.9	37.8
**7D**	11	39	28	2.5	14.0
**Overall**	**Tot. 551**	**Tot. 2711**	**Tot. 2447.4**	**Ave. 4.44**	**Ave. 37.8**

*Not including the gap between linkage groups, when chromosomes were mapped as multiple linkage groups
