# Supplementary material for: A QTL on the short arm of wheat (Triticum aestivum L.) chromosome 3B affects the stability of grain weight in plants exposed to a brief heat shock early in grain filling
Source: BMC Plant Biol. 2016 Apr 22;16:100. doi: 10.1186/s12870-016-0784-6 (PMC4841048; doi:10.1186/s12870-016-0784-6)
Supplement: Additional file 10: Figure S2. — QTL detected for heat susceptibility (HSI) and absolute trait values in the Drysdale × Waagan doubled haploid population. The numbers to the left of each molecular marker linkage group indicate cM distances from the top. Colored boxes represent peaks of QTL effects, defined in each case by the markers with –Log10(p) test statistic values within 1.5 of the peak value. Blue: QTL for HSIs; black: QTL for DTA and plant height; green, red, and brown: QTL detected for the absolute trait values under control, heat, and both control and heat conditions, respectively. Solid and hashed bars indicate QTL detected in both experiments or in one experiment only, except for single-experiment effects where the bar was too short to show hashing: QTL2-all-traits, QTL11-HChlR27, QTL11-ChlR27-heat, QTL11-HI-control and QTL29-FL-control. DTA, days from sowing to anthesis; DTM, days from sowing to maturity; GFD, grain-filling duration defined as days from anthesis to 95 % spike senescence; FLSe, days from anthesis to 95 % flag leaf senescence; GWS, grain weight spike−1; GNS, grain number spike−1; SGW, single grain weight; ShW, shoot dry weight; PH, plant height; ChlC10DAA, chlorophyll content 10 days after anthesis; ChlC13DAA, chlorophyll content 13 days after anthesis; AUSC, area under the SPAD curve; ChlR13, rate of chlorophyll change between 10 and 13 days after anthesis; ChlR27, rate of chlorophyll change based on the linear regression of the measurements, at 10, 13 and 27 days after anthesis; FL, flag leaf length; FW, flag leaf width; HI, harvest index. An ‘R’ next to a marker indicates that it represents a group of genetically redundant markers. (PDF 168 kb) [file 12870_2016_784_MOESM10_ESM.pdf]

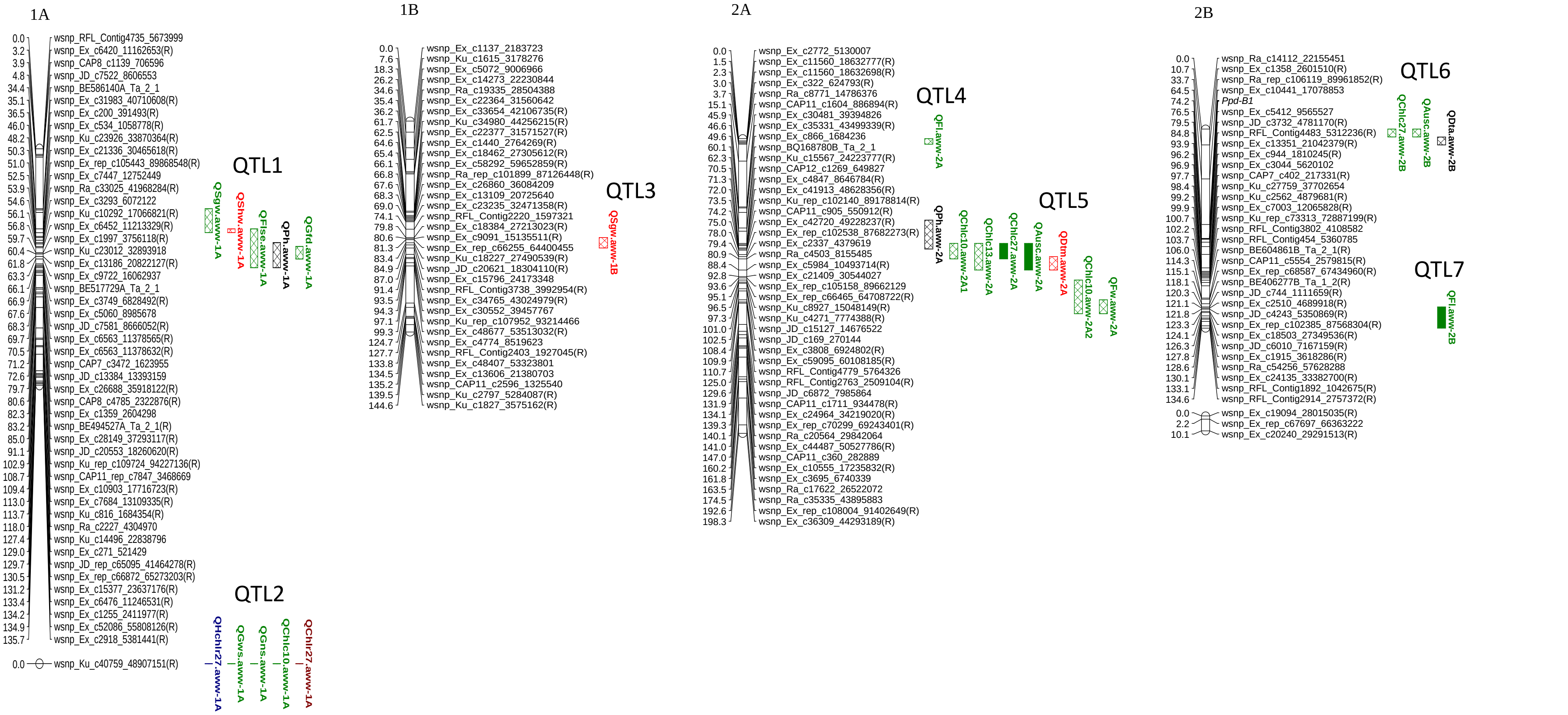

Figure S2. QTL detected for heat susceptibility (HSI) and absolute trait values in the Drysdale × Waagan doubled haploid population. The numbers to the left of each molecular marker linkage group indicate cM distances from the top. Colored boxes represent peaks of QTL effects, defined in each case by the markers with $-Log_{10}(p)$ test statistic values within 1.5 of the peak value. Blue: QTL for HSIs; black: QTL for DTA and plant height; green, red, and brown: QTL detected for the absolute trait values under control, heat, and both control and heat conditions, respectively. Solid and hashed bars indicate QTL detected in both experiments or in one experiment only, except for single-experiment effects where the bar was too short to show hashing: QTL2-all-traits, QTL11-HChlR27, QTL11-ChlR27-heat, QTL11-HI-control and QTL29-FL-control. DTA, days from sowing to anthesis; DTM, days from sowing to maturity; GFD, grain-filling duration defined as days from anthesis to 95% spike senescence; FLSe, days from anthesis to 95% flag leaf senescence; GWS, grain weight spike^{-1}; GNS, grain number spike^{-1}; SGW, single grain weight; ShW, shoot dry weight; PH, plant height; ChlC10DAA, chlorophyll content 10 days after anthesis; ChlC13DAA, chlorophyll content 13 days after anthesis; AUSC, area under the SPAD curve; ChlR13, rate of chlorophyll change between 10 and 13 days after anthesis; ChlR27, rate of chlorophyll change based on the linear regression of the measurements, at 10, 13 and 27 days after anthesis; FL, flag leaf length; FW, flag leaf width; HI, harvest index. An 'R' next to a marker indicates that it represents a group of genetically redundant markers.

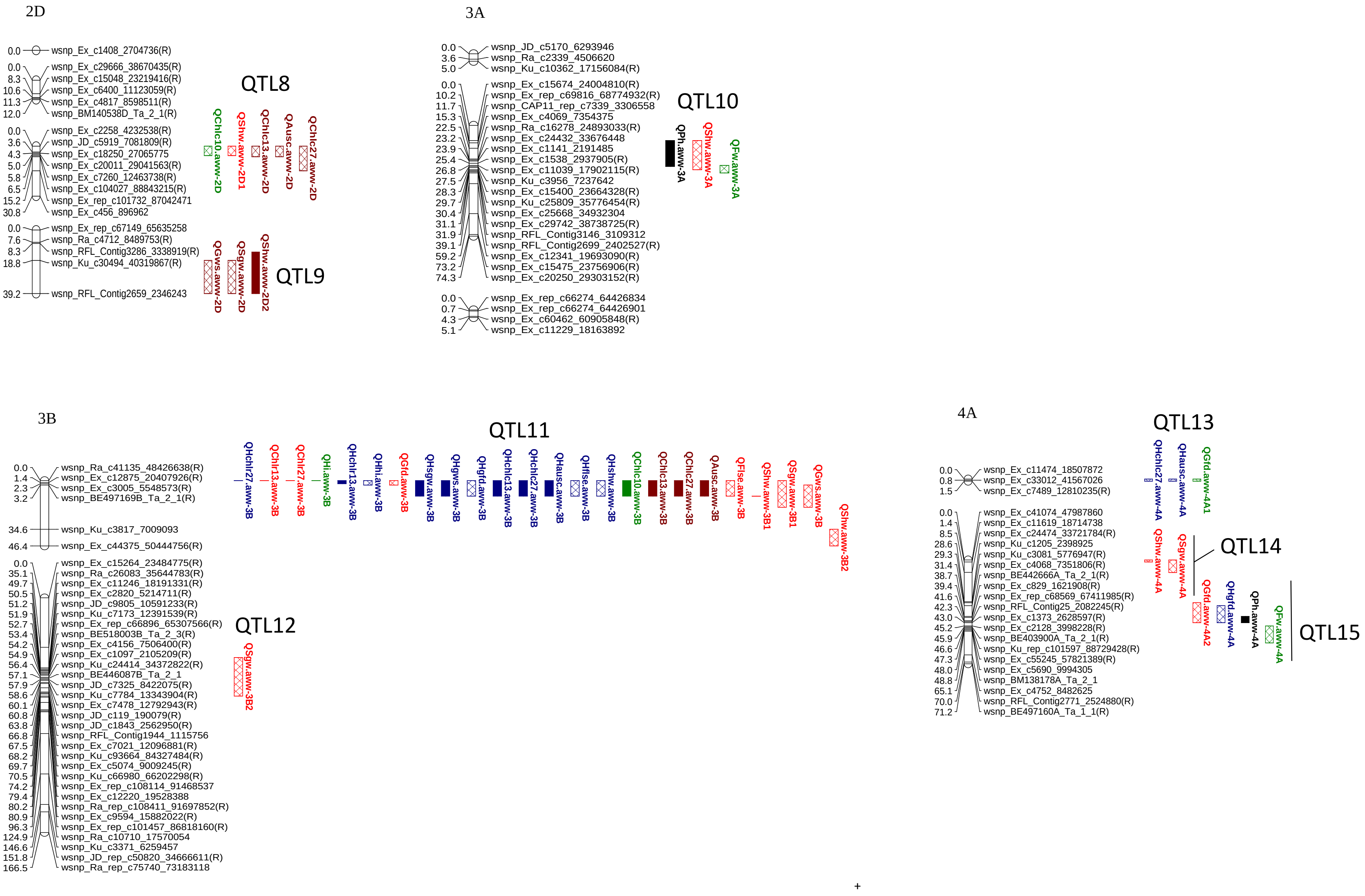

Figure S2 (continued)

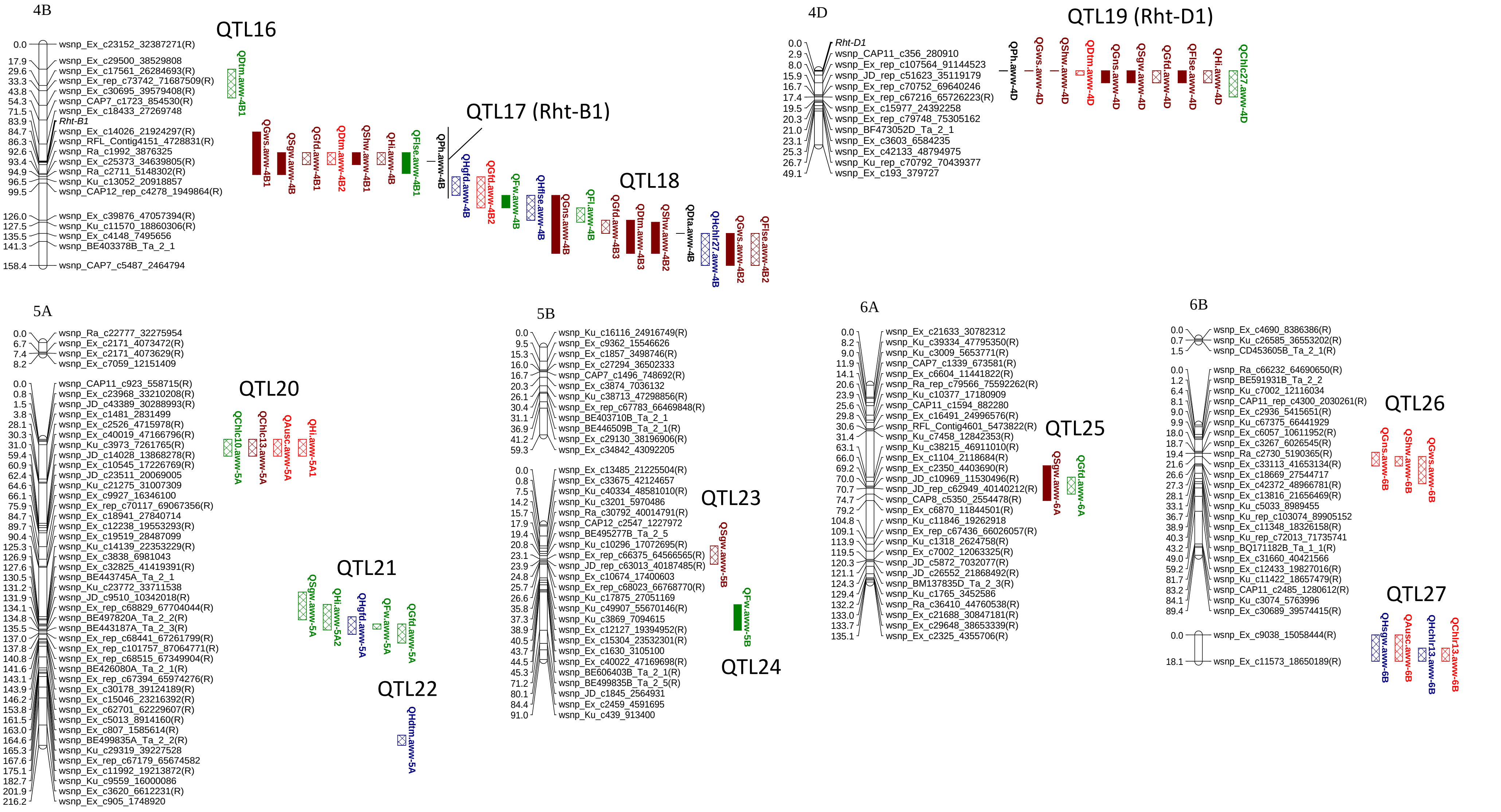

Figure S2 (continued)

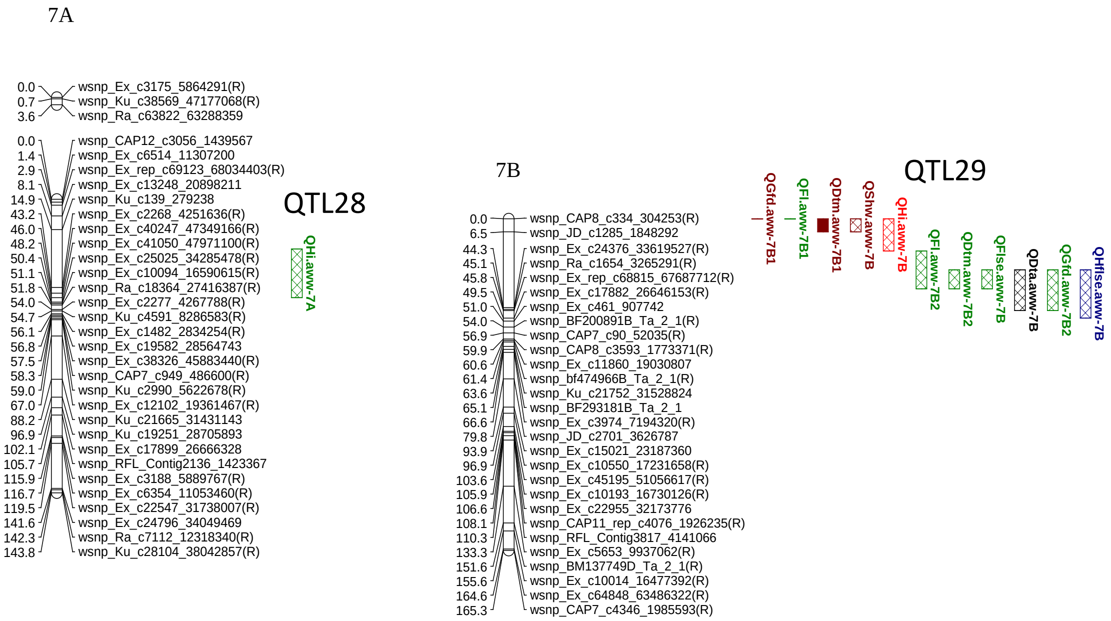

Figure S2 (continued)
