# Supplementary material for: A QTL on the short arm of wheat (Triticum aestivum L.) chromosome 3B affects the stability of grain weight in plants exposed to a brief heat shock early in grain filling
Source: BMC Plant Biol. 2016 Apr 22;16:100. doi: 10.1186/s12870-016-0784-6 (PMC4841048; doi:10.1186/s12870-016-0784-6)
Supplement: Additional file 11: Figure S3. — Heat-plot for final Drysdale × Waagan genetic map of 551 loci. Represented are recombination fractions (upper-left half of figure) and LOD scores for linkage (lower-right half of figure) for all pairs of genetically non-redundant markers. Markers are arranged in order and by chromosome or chromosome fragment, from chromosome 1A (left) to 7D (right). With progression through the colour series blue-green-yellow-red, LOD score increases and recombination fraction decreases. Alignment of the red signals along the diagonal indicates that the marker orders are largely correct. (PDF 404 kb) [file 12870_2016_784_MOESM11_ESM.pdf]

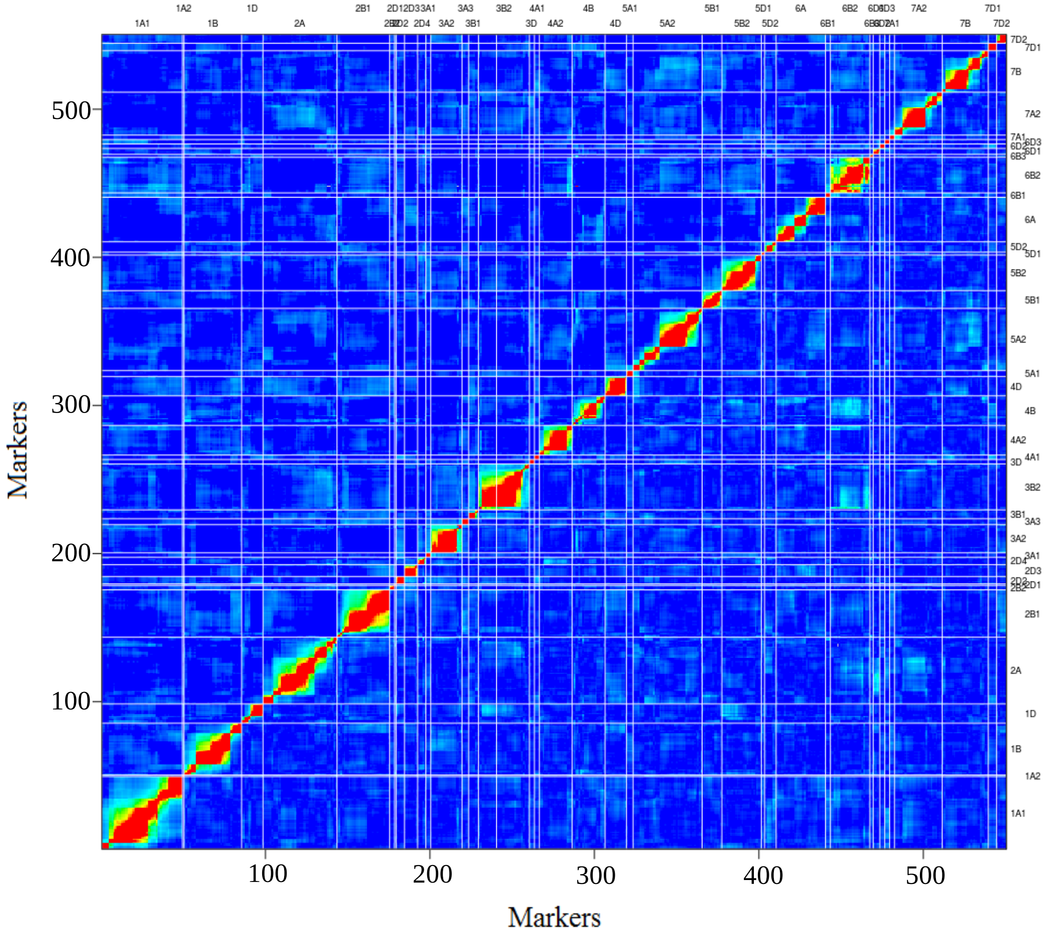

Figure S3. Heat-plot for final Drysdale x Waagan genetic map of 551 loci. Represented are recombination fractions (upper-left half of figure) and LOD scores for linkage (lower-right half of figure) for all pairs of genetically non-redundant markers. Markers are arranged in order and by chromosome or chromosome fragment, from chromosome 1A (left) to 7D (right). With progression through the colour series blue-green-yellow-red, LOD score increases and recombination fraction decreases. Alignment of the red signals along the diagonal indicates that the marker orders are largely correct.
